# Efficacy of Tofacitinib in Refractory Axial Spondylarthritis: A Dose-Escalation Study

**DOI:** 10.7759/cureus.94023

**Published:** 2025-10-07

**Authors:** Abdul Mahin Tazbir, Mohammad Abul Kalam Azad, Abul Khair Ahmedullah, M Masudul Hassan, Fahmida Nishu, Md Atiqur Rahman, Toufiqe E Ealahi, Md Nazrul Islam

**Affiliations:** 1 Rheumatology, Enam Medical College Hospital, Dhaka, BGD; 2 Rheumatology, Bangladesh Medical University, Dhaka, BGD; 3 Psychiatry, Bangladesh Medical University, Dhaka, BGD; 4 Internal Medicine, Central Police Hospital, Dhaka, BGD; 5 Internal Medicine, Barishal Medical College Hospital, Barishal, BGD

**Keywords:** axial spondyloarthritis, dose escalation, jak inhibitor, rhinitis, tofacitinib

## Abstract

Objectives

The objective was to evaluate the efficacy and safety of escalating tofacitinib from 10 mg to 15 mg daily in NSAID-refractory axial spondyloarthritis (ax-SpA) patients with inadequate 12-week response to 10 mg.

Methods

This was a pragmatic, open-label, single-arm, dose-escalation study (24 weeks). 101 patients were enrolled (ITT); 88 completed both week-12 and week-24 assessments (evaluable). All started tofacitinib 10 mg daily. At week 12, those achieving ASDAS-CRP (Ankylosing Spondylitis Disease Activity Score - C-reactive protein) major improvement (MI; Δ≥2.0) continued 10 mg; non-responders escalated to 15 mg. Outcomes included ASDAS-CRP (Clinically Important Improvement (CII)/MI/inactive disease), Assessment of SpondyloArthritis International Society criteria (ASAS 20/40), Bath Ankylosing Spondylitis Disease Activity Index (BASDAI), Bath Ankylosing Spondylitis Functional Index (BASFI), Bath Ankylosing Spondylitis Metrology Index (BASMI), Ankylosing Spondylitis Quality of Life (ASQoL), and 36-Item Short-Form Health Survey (SF-36); safety was monitored per Common Terminology Criteria for Adverse Events (CTCAE) v5.0.

Results

Of the evaluable cohort (n=88), 57 (64.8%) achieved MI at week 12 and continued 10 mg; 31 (35.2%) escalated to 15 mg. By week 24, mean ASDAS-CRP improved from 4.4→1.7 (10 mg) and 4.0→2.2 (15 mg). In the escalation subgroup, ASDAS-CRP improved 3.0 ± 0.7→2.2 ± 0.7, BASDAI 3.9 ± 1.3→2.9 ± 1.2, and ASAS 20/40 responses rose to 93.5%/45.2%. Six patients discontinued due to AEs (5 transaminitis at ≤10 mg; 1 breast tumour). Rhinitis was more frequent with 15 mg (58.1% vs 31.6%). No TB, VTE, MACE, or deaths occurred.

Conclusions

In patients not meeting 12-week targets on 10 mg, escalation to 15 mg yielded additional clinically meaningful improvement with a safety profile generally comparable to 10 mg. Selective escalation may be a feasible treat-to-target strategy where alternatives are limited.

## Introduction

Spondyloarthritis (SpA) comprises a group of inflammatory rheumatic diseases, broadly classified into axial SpA (ax-SpA) and peripheral SpA [1]. The prevalence of SpA ranges from 0.03% in India [2] to 0.2% [3], and up to 1.2% in Bangladesh, and a male-to-female ratio is approximately 2:1 [4]. Axial SpA primarily manifests as inflammatory low back pain (LBP), with patients further categorized into non-radiographic axial SpA (nr-axSpA) and radiographic axial SpA, depending on sacroiliac joint radiological findings [5].

Delayed diagnosis of ax-SpA often leads to functional impairment, work disability, and a significant reduction in quality of life [6]. It also adversely impacts psychosocial well-being [7]. According to current treatment guidelines, non-steroidal anti-inflammatory drugs (NSAIDs) are the first-line therapy. If one NSAID fails, an alternative is prescribed. Patients who show a poor response to two or more NSAIDs over at least two weeks at optimal dosages are considered NSAID-refractory [8]. In such cases, targeted therapies such as tumour necrosis factor inhibitors (TNFi), interleukin-17 inhibitors (IL-17i), and Janus kinase inhibitors (JAKi), including tofacitinib, upadacitinib, and filgotinib, are considered [9]. Tofacitinib, a JAK inhibitor, has emerged as a promising treatment option in NSAID-refractory ax-SpA. However, in Bangladesh, access to biologic therapies remains limited due to high costs, making guideline-directed care challenging.

Tofacitinib, being relatively more affordable, is more commonly used in this context. A phase 3 trial demonstrated a 26% major improvement in Ankylosing Spondylitis Disease Activity Score - CRP (ASDAS-CRP) scores with tofacitinib 10 mg daily [10]. Nevertheless, a substantial number of patients remained unresponsive at this dosage, suggesting a potential benefit from escalating the dose to 15 mg daily. This highlights the need for further research into the efficacy and safety of tofacitinib dose escalation in ax-SpA. Although the U.S. FDA has issued warnings against the use of tofacitinib 20 mg daily due to safety concerns [11], the use of 15 mg remains underexplored in ax-SpA.

Tofacitinib selectively inhibits Janus kinases (JAK 1, 2, 3, and tyrosine kinase 2 (TYK2)), thereby blocking the Janus Kinase-Signal Transducer and Activator of Transcription (JAK-STAT) signaling pathway and downregulating pro-inflammatory cytokine production. It inhibits the expression of IL-17, IL-21, IL-23, and interferon gamma (IFN-γ) by CD4+ T cells [12], and acts on dendritic cells to suppress inflammation and joint damage. Tofacitinib is already an established therapy for rheumatoid arthritis (RA) [13], psoriatic arthritis (PsA) [14], ulcerative colitis (UC) [15], polyarticular juvenile idiopathic arthritis [16], and axial SpA [17].

A significant proportion of patients with chronic inflammatory low back pain in Bangladesh are ultimately found to have axial spondyloarthritis (axSpA), but only about 12-15% achieve an adequate response on NSAIDs alone [18]. Biologics like TNFi or IL-17i are often unaffordable, making tofacitinib - an oral, cost-effective JAK inhibitor - a more accessible option.

While tofacitinib 10 mg is commonly used, nearly one-third of patients show inadequate response, and switching to other therapies is often not feasible due to cost. Though 20 mg has known safety risks, 15 mg has shown no additional adverse effects in RA and UC trials but remains unstudied in ax-SpA [19].

This study aims to evaluate the clinical outcomes, including ASDAS-CRP response, and adverse effects of tofacitinib 15 mg daily in patients with refractory ax-SpA who have failed to respond to 10 mg daily. Additionally, it will explore factors associated with treatment failure at lower doses.

## Materials and methods

Patient selection

Adults (≥18 years) with axial spondylarthritis (axSpA) were eligible if they had tried ≥2 courses of NSAIDs at optimal doses for ≥1 month with inadequate or partial response and had high/very high disease activity (ASDAS-CRP >2.1). Key exclusions were prior targeted synthetic disease-modifying antirheumatic drugs (tsDMARDs) or ** **biologic disease-modifying antirheumatic drugs (bDMARDs); recent or active infection (including tuberculosis); anemia (hemoglobin <9 g/dL); leukopenia (WBC <4,000/mm³), neutropenia (<1,000/mm³) or thrombocytopenia (<100,000/mm³); live vaccines within 3 months before first dose; estimated glomerular filtration rate (eGFR) <60 mL/min/1.73 m²; alanine aminotransferase (ALT) >3× upper limit of normal; history of thromboembolism, deep venous thrombosis, diverticulitis or malignancy; and pregnancy or breastfeeding.

We enrolled 101 participants (intention-to-treat (ITT)), all starting tofacitinib 10 mg once daily. Detailed history, examination, and baseline labs were recorded at initiation. Bath Ankylosing Spondylitis Functional Index (BASFI), Bath Ankylosing Spondylitis Metrology Index (BASMI), Maastricht Ankylosing Spondylitis Enthesitis Score (MASES), Bangla Health Assessment Questionnaire for Disability Index (B-HAQ-DI), and Bangla version of the 36-Item Short-Form Health Survey (B-SF-36) were assessed at baseline, week 12, and week 24. ASDAS-CRP (clinically important improvement, major improvement, and inactive disease) and Assessment of SpondyloArthritis International Society criteria (ASAS 20/40) were assessed at weeks 4, 12, and 24. Participants were scheduled for follow-up at weeks 4, 12, and 24.

**Figure 1:**
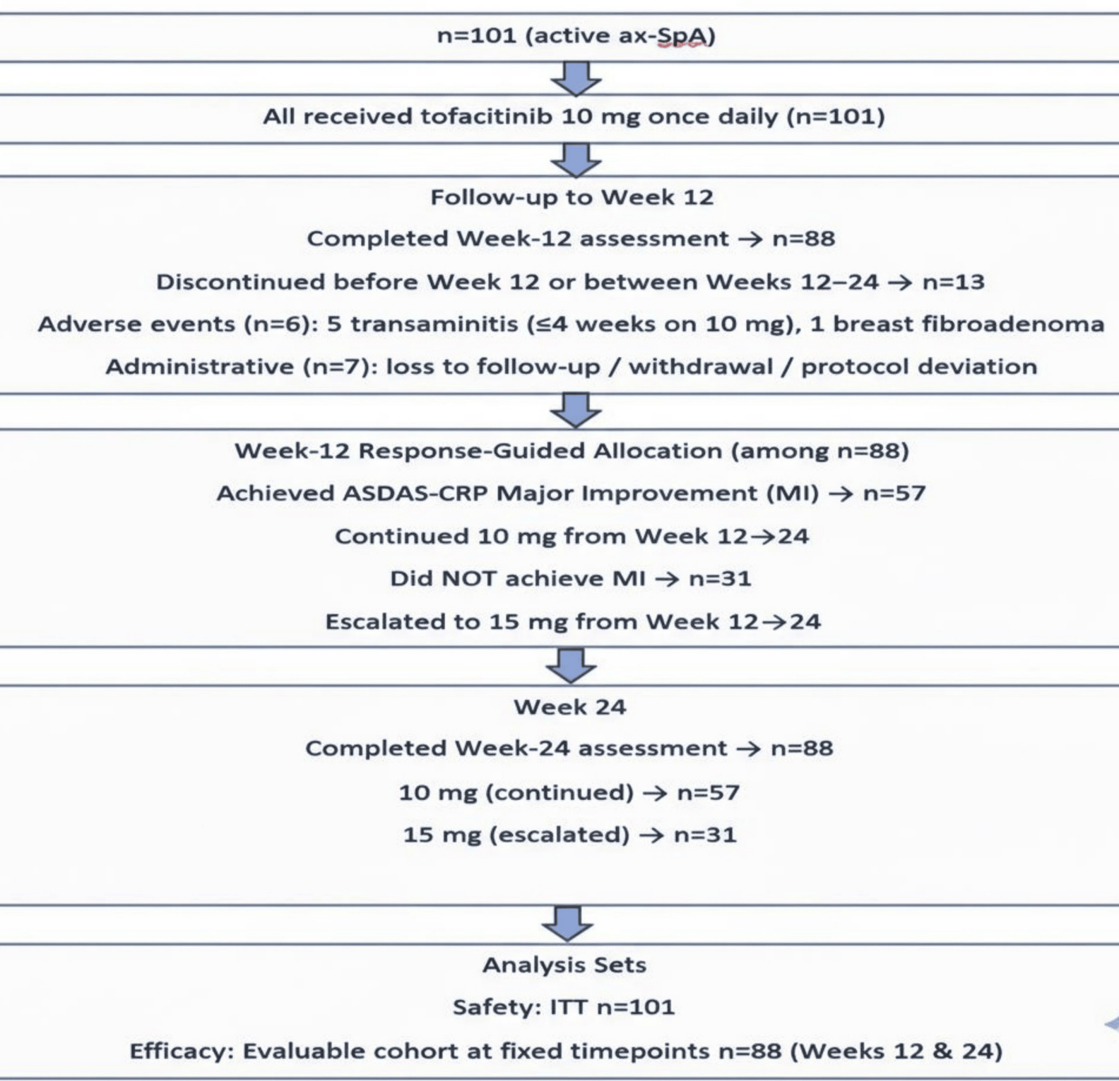
CONSORT flow diagram of enrollment, response-guided allocation at week 12, follow-up, and analysis sets CONSORT: CONsolidated Standards Of Reporting Trials; axSpA: axial spondyloarthritis; ASDAS-CRP: Ankylosing Spondylitis Disease Activity Score - C-reactive protein; ITT: intention-to-treat

At week 12, dosing was response-guided: participants who achieved ASDAS-CRP major improvement (MI) continued 10 mg; those without MI escalated to 15 mg from week 12 to 24. Efficacy analyses at weeks 12 and 24 used the evaluable cohort; safety analyses used the ITT set. Of 101 enrolled, 88 completed the week-12 and week-24 assessments and comprised the evaluable cohort. Thirteen discontinued before week 12 or between weeks 12-24: six due to adverse events (five cases of transaminitis within the first 4 weeks on 10 mg; one breast fibroadenoma) and seven for administrative reasons (loss to follow-up, withdrawal, or protocol deviation). At week 12, 57 participants achieved MI and continued 10 mg, while 31 did not achieve MI and escalated to 15 mg. The study continued to week 24; non-responders at week 24 were switched to alternative therapy (Figure 1).

Study design

This was an open-label, single-arm, non-randomized, pragmatic dose-escalation study conducted at the Department of Rheumatology, Bangabandhu Sheikh Mujib Medical University (BSMMU) (January 2023-January 2024; IRB approval BSMMU/2022/4139; ClinicalTrials.gov NCT06310057). All participants started tofacitinib 10 mg once daily. At week 12, those achieving ASDAS-CRP major improvement (Δ ≥ 2.0) continued 10 mg, while others escalated to 15 mg once daily for weeks 12-24. No allocation concealment or blinding was used. All participants provided written informed consent.

Adults (≥18 y) with ax-SpA (nr-axSpA or radiographic) and ASDAS-CRP > 2.1 after ≥2 NSAID courses at optimal doses for ≥1 month were eligible. Key exclusions: prior ts/bDMARDs; active/recent infection (including TB); hemoglobin (Hgb) < 9 g/dL; WBC < 4,000/mm³; absolute neutrophilic count (ANC) < 1,000/mm³; platelets < 100,000/mm³; eGFR < 60 mL/min/1.73 m²; alanine aminotransferase (ALT) > 3×ULN; thromboembolism; diverticulitis; malignancy; pregnancy/lactation; live vaccines ≤3 months.

All patients initiated tofacitinib 10 mg once daily at baseline. At week 12, patients with ASDAS-CRP MI (Δ≥2.0) continued 10 mg; those without MI escalated to 15 mg once daily for weeks 12-24. The 12-week decision point aligns with treat-to-target recommendations and early JAK-inhibitor response kinetics. Escalation to 15 mg was selected as a feasibility/safety compromise in a resource-limited setting, avoiding the safety concerns reported with total daily exposures of 20 mg.

Adverse events (AEs) were captured at each visit and during interim phone calls. Common Terminology Criteria for Adverse Events (CTCAE) v5.0 was used for grading; seriousness per International Council for Harmonisation of Technical Requirements for Pharmaceuticals for Human Use (ICH) criteria; causality via World Health Organization - Uppsala Monitoring Centre (WHO-UMC) categories.

Stopping rules were ALT/aspartate aminotransferase (AST) >3×ULN with symptoms or >5×ULN without symptoms; severe infection; pregnancy; or any serious adverse events (SAE) deemed related.

Labs included complete blood count (CBC), erythrocyte sedimentation rate (ESR)/C-reactive protein (CRP), creatinine, ALT/AST, fasting lipid panel at baseline, week 12, week 24 (additional testing as indicated). Tuberculosis (TB) screening (Tuberculin Skin Test / Interferon-Gamma Release Assay (TST/IGRA)) and chest radiography were performed pre-treatment.

Sample Size Calculation

We based the sample size calculation on the assumption that the proportion of patients achieving minimal improvement (MI) in ASDAS-CRP would be approximately 30% in the 10 mg group and 50% in the escalation group (15 mg). Using a two-sided significance level (α) of 0.05 and aiming for 80% power to detect this difference between groups, we calculated that approximately 90 participants per group would be required.

To account for potential attrition, including loss to follow-up or missing data, we inflated the sample size by 10%, resulting in a target enrollment of 101 participants per group.

The intention-to-treat (ITT) cohort comprised all 101 enrolled. The evaluable cohort included 88 with complete week-12 and week-24 data. Primary analyses used the evaluable cohort; sensitivity analyses used non-responder imputation (NRI) for binary endpoints (ASDAS states, ASAS20/40) and Last Observation Carried Forward (LOCF) for continuous measures.

At baseline, detailed history, physical examination, and laboratory investigations were performed. Functional and quality of life indices, including BASFI [20], BASMI, MASES, B-HAQ-DI [21], and B-SF-36 [22], were assessed at baseline, 12 weeks, and 24 weeks.

Disease activity was evaluated using indices such as ASDAS-CRP, including clinically important improvement, major improvement, and inactive disease states, alongside ASAS 20 and ASAS 40. These were recorded at weeks 4, 12, and 24.

Laboratory investigations included CBC, ESR, CRP, serum glutamic-pyruvic transaminase (SGPT), serum creatinine, fasting lipid profile, tuberculosis screening tests (TST/IGRA), X-rays of sacroiliac joints, human leukocyte antigen (HLA)-B27 typing, and chest X-ray (posteroanterior view).

At the 12th week, patients who achieved ASDAS major improvement (≥ 2.0) continued on tofacitinib 10 mg, whereas those who did not meet this criterion had their dose escalated to tofacitinib 15 mg.

Week 12 was chosen as the decision point based on treat-to-target guidance and observed early JAK-inhibitor kinetics. We selected 15 mg once daily as a safety-conscious escalation (avoiding total daily exposure of 20 mg that raised safety concerns), while aiming to recapture non-responders where alternatives are limited.

Primary Outcome

The primary outcome was the proportion achieving ASDAS-CRP MI (Δ≥2.0) at week 24 in the escalation subgroup.

Key Secondary Outcomes

The key secondary outcomes were ASDAS-CRP CII (Δ≥1.1), inactive disease (<1.3), ASAS20/40, BASDAI, BASFI, BASMI, ASQoL, SF-36 PCS/MCS; time-to-MI (Kaplan-Meier). Assessments at baseline, week 12, and week 24 (plus week 4 symptom scales).

Statistical analysis

Primary analyses used the evaluable cohort (n=88). Sensitivity analyses employed non-responder imputation (binary endpoints) and LOCF (continuous outcomes); results were directionally consistent. Continuous variables are presented as mean ± SD. Between-group comparisons were performed using the unpaired t-test or the Mann-Whitney U test, as appropriate. Within-group changes were assessed using the paired t-test; we report Δ means with 95% confidence intervals (CIs), Cohen’s d, and the standardized response mean (SRM). Between-group differences in change (ΔΔ) are presented as the mean difference with 95% CI and Hedges g. Categorical outcomes were analyzed using the χ² test or Fisher’s exact test. Prespecified subgroups included age (median split), sex, disease duration (median split), and baseline ASDAS-CRP (<3.5/≥3.5); subgroup effects were tested via interaction terms. Two-sided p-values <0.05 were considered statistically significant. All analyses were conducted using SPSS version 25 (IBM Corp., Armonk, USA).

## Results

Of 101 enrolled (ITT), 88 completed week-12 and week-24 assessments (evaluable). Thirteen patients discontinued: six due to AEs (5 transaminitis ≤4 weeks on 10 mg; 1 new breast tumour) and seven due to administrative reasons (loss to follow-up/withdrawal/protocol deviation).

The 88 participants who remained on 10 mg and achieved MI were younger (31.5 ± 9.7 vs 38.3 ± 9.9 years; p=0.003). The 15 mg group had more married individuals (83.9% vs 57.9%; p=0.01), while education and occupation showed non-significant trends; sex distribution and residence were similar. Clinical background and treatment history were otherwise comparable, except for greater sulfasalazine use at baseline in the 15 mg group (90.3% vs 71.9%; p=0.045) (Table 1).

**Table 1:** Comparison of baseline characteristics of patients with 10mg Tofacitinib (achieved ASDAS CRP major improvement criteria at 12th week, n=57) and 15mg Tofacitinib (failed to achieve ASDAS CRP major improvement criteria at 12th week, n=31) Statistics: ^*^Categorical variables compared with χ² test; ^**^continuous variables with independent t-test. Values are n (%) or mean ± SD. ILBP = Inflammatory back pain; SSZ = Sulfasalazine; MTX = Methotrexate; HCQ = Hydroxychloroquine; DM = Diabetes mellitus; HTN = Hypertension; IHD = Ischemic heart disease; IDA = Iron-deficiency anemia; BMI = Body mass index; SD = Standard deviation.

Characteristic		Tofacitinib 10 mg, Major improvement achieved (n=57)	Tofacitinib 15 mg, Major improvement not achieved (n=31)	p-value
Sex,	Male/Female	41 (71.9%)/16 (28.1%)	17 (54.8%)/14 (45.2%)	0.106*
Marital status	Married	33 (57.9%)	26 (83.9%)	0.01*
	Unmarried/others	24 (42.1%)/0 (–)	4 (12.9%)/1 (3.2%)	
Education	Illiterate	6 (10.5%)	4 (12.9%)	0.096*
	Up to class 5	8 (14.0%)	4 (12.9%)	-
	Up to class 8	5 (8.8%)	10 (32.3%)	-
	SSC/HSC	10 (17.5%)/16 (28.1%)	6 (19.4%)/5 (16.1%)	--
	Graduate/Postgraduate	10 (17.5%)/2 (3.5%)	2 (6.5%)/0 (–)	
Occupation	Housewife	8 (14.0%)	13 (41.9%)	0.065*
	Business	4 (7.0%)	2 (6.5%)	-
	Service	23 (40.4%)	5 (16.1%)	-
	Student	11 (19.3%)	4 (12.9%)	-
	Agriculture	1 (1.8%)	0 (–)	-
	Unemployed	7 (12.3%)	4 (12.9%)	-
	Others	3 (5.3%)	3 (9.7%)	-
Residence	Urban/Rural	28 (49.1%)/29 (50.9%)	16 (51.6%)/15 (48.4%)	0.823**
Age, years (mean ± SD)		31.5 ± 9.7	38.3 ± 9.9	0.003**
Height, cm (mean ± SD)		161.0 ± 8.9	159.0 ± 6.6	0.23**
Weight, kg (mean ± SD)		60.5 ± 12.5	61.7 ± 14.1	0.683**
BMI, kg/m² (mean ± SD)		23.6 ± 4.7	24.6 ± 5.1	0.374**
First symptom at onset	Low back pain	22 (38.6%)	12 (38.7%)	0.696*
	Buttock pain	3 (5.3%)	3 (9.7%)	-
	Lower limb arthritis	25 (43.9%)	10 (32.3%)	-
	Upper limb arthritis	2 (3.5%)	1 (3.2%)	-
	Enthesitis	5 (8.8%)	5 (16.1%)	-
SpA Subtype	Ankylosing spondylitis (AS)	56 (98.2%)	30 (96.8%)	0.303*
	Psoriatic arthritis (PsA)	0 (–)	1 (3.2%)	-
	IBD-associated arthritis	1 (1.8%)	0 (–)	-
Family history of SpA	Positive/ Negative	19 (33.3%)/38 (66.7%)	8 (25.8%)/23 (74.2%)	0.465*
HLAB27	Negative	3 (5.3%)	2 (6.5%)	0.797*
	Positive	30 (52.6%)	14 (45.2%)	-
	Not tested	24 (42.1%)	15 (48.4%)	-
Sacroiliitis diagnosed	No	6 (10.5%)	2 (6.5%)	0.751*
	Yes, with X-rays	50 (87.7%)	28 (90.3%)	--
	Yes, with MRI	1 (1.8%)	1 (3.2%)	-
Hip involvement	No	45 (78.9%)	22 (71.0%)	0.685*
	Yes, unilateral (right)	4 (7.0%)	3 (9.7%)	-
	Yes, unilateral (left)	3 (5.3%)	1 (3.2%)	-
	Yes, bilateral	5 (8.8%)	5 (16.1%)	-
Disease duration, years (mean ± SD)		8.1 ± 5.5	10.1 ± 6.8	0.153**
Age at onset, years (mean ± SD)		23.7 ± 8.5	28.3 ± 11.4	0.055**
Age at diagnosis, years (mean ± SD)		28.4 ± 8.5	35.0 ± 10.1	0.003**
ILBP, duration, years (mean ± SD)		6.8 ± 5.2	8.1 ± 5.9	0.312**
Gap from work due to disease, months (mean ± SD)		4.8 ± 7.0	9.6 ± 16.7	0.129*
SSZ at baseline,	Yes/ No	41 (71.9%)/16 (28.1%)	28 (90.3%)/3 (9.7%)	0.045*
MTX at baseline	Yes/No	11 (19.3%)/46 (80.7%)	5 (16.1%)/26 (83.9%)	0.713*
HCQ	Yes/No	1 (1.8%)/56 (98.2%)	0 (–)/ 31 (100.0%)	0.99*
Leflunomide at baseline	Yes/No	1 (1.8%)/55 (98.2%)	0 (–)/31 (100.0%)	0.99*
DM	Yes/No	5 (8.8%)/52 (91.2%)	4 (12.9%)/27 (87.1%)	0.715*
Hypertension	Yes/No	4 (7.0%)/53 (93.0%)	5 (16.1%)/26 (83.9%)	0.269*
Hypothyroidism	Yes/ No	2 (3.5%)/55 (96.5%)	2 (6.5%)/29 (93.5%)	0.611*
Asthma		57 (100.0%)	31 (100.0%)	0.99*
IHD	Yes/No	0 (–)/57 (100.0%)	2 (6.5%)/29 (93.5%)	0.121*
IDA	Yes/No	0 (–)/57 (100.0%)	2 (6.5%)/29 (93.5%)	0.121*
Dyslipidemia	Yes/ No	13 (22.8%)/44 (77.2%)	9 (29.0%)/22 (71.0%)	0.519*

Baseline symptoms, function, quality of life (QoL), and labs. Pain scores (overall, axial, night), morning stiffness, fatigue, BASDAI/BASFI/BASMI, ASQoL, and routine chemistries were comparable between groups (p>0.05). The 10 mg group started with higher inflammatory activity: ASDAS-CRP 4.4 ± 0.7 vs 4.0 ± 0.9 (p=0.02), ASDAS-ESR 4.3 ± 1.0 vs 3.9 ± 0.7 (p=0.045), CRP 47.1 ± 45.5 vs 25.1 ± 38.5 mg/L (p=0.019), and ESR 56.9 ± 42.1 vs 33.1 ± 24.1 mm/hr (p=0.001) (Table 2).

**Table 2:** Comparison of baseline disease characteristics, laboratory, disease indices and quality of life between subjects with 10 mg tofacitinib (achieved ASDAS-CRP major improvement criteria at 12th week, n=57) and 15 mg tofacitinib (failed to achieve ASDAS-CRP major improvement criteria at 12th week, n=31). ASDAS-CRP = Ankylosing Spondylitis Disease Activity Score - C-reactive protein; ASDAS-ESR = Ankylosing Spondylitis Disease Activity Score–erythrocyte sedimentation rate; ASQoL = Ankylosing Spondylitis Quality of Life; BASDAI = Bath Ankylosing Spondylitis Disease Activity Index; BASFI = Bath Ankylosing Spondylitis Functional Index; BASMI = Bath Ankylosing Spondylitis Metrology Index; NRS = Numerical rating scale; PGA = Patient Global Assessment; CRP = C-reactive protein; ESR = Erythrocyte sedimentation rate; ALT/SGPT = Alanine aminotransferase/serum glutamic pyruvic transaminase; HDL-C = High-density lipoprotein cholesterol; LDL-C = Low-density lipoprotein cholesterol; SD = Standard deviation.

Characteristic	Tofacitinib 10 mg, Major improvement achieved (n=57)	Tofacitinib 15 mg, Major improvement not achieved (n=31)	p-value
Pain (NRS 0–10), baseline	7.7 ± 1.0	7.7 ± 1.2	0.822
Axial pain (0–10), baseline	7.4 ± 1.1	7.5 ± 1.3	0.683
Spine pain at night (0–10), baseline	7.3 ± 1.2	7.7 ± 1.2	0.127
Morning spinal stiffness, minutes (baseline)	59.2 ± 27.2	70.3 ± 37.3	0.15
Fatigue (0–10), baseline	6.5 ± 1.2	6.9 ± 1.2	0.181
ASDAS-CRP, baseline	4.4 ± 0.7	4.0 ± 0.9	0.02
ASDAS-ESR, baseline	4.3 ± 1.0	3.9 ± 0.7	0.045
BASDAI, baseline	5.7 ± 1.1	6.2 ± 1.2	0.095
BASFI, baseline	7.6 ± 0.8	7.7 ± 0.9	0.835
BASMI (linear), baseline	2.6 ± 1.4	2.9 ± 1.6	0.351
ASQoL, baseline	15.5 ± 2.2	15.3 ± 3.4	0.754
CRP, mg/L (baseline)	47.1 ± 45.5	25.1 ± 38.5	0.019
ESR, mm/hr (baseline)	56.9 ± 42.1	33.1 ± 24.1	0.001
Hemoglobin, g/dL (baseline)	11.7 ± 2.6	11.9 ± 1.1	0.552
Serum creatinine, mg/dL (baseline)	0.8 ± 0.2	0.8 ± 0.2	0.649
ALT/SGPT, U/L (baseline)	24.1 ± 17.9	22.0 ± 12.4	0.518
Total cholesterol, mg/dL (baseline)	165.3 ± 46.2	159.2 ± 38.5	0.508
Triglycerides, mg/dL (baseline)	211.0 ± 526.4	170.6 ± 105.4	0.578
HDL-cholesterol, mg/dL (baseline)	42.1 ± 9.8	41.7 ± 9.8	0.856
LDL-cholesterol, mg/dL (baseline)	105.3 ± 35.9	96.4 ± 34.7	0.272

Change favored the 10 mg group for ASDAS-CRP, ASQoL, BASDAI, and BASFI at both intervals (all ΔΔ 95% CIs exclude 0; effects moderate-large). Example: ΔΔASDAS-CRP 0→12 = −1.44 (95% CI −1.70 to −1.19) and 0→24 = −0.80 (−1.19 to −0.41). BASMI differences were not significant (CIs include 0) (Table 3).

**Table 3:** Between-group differences in change (ΔΔ) from baseline to weeks 12 and 24 for ASDAS-CRP, ASQoL, BASDAI, BASFI, and BASMI (10 mg vs 15 mg) Δ = Change from baseline; ΔΔ = Between-group difference in change (10 mg − 15 mg); ASDAS-CRP = Ankylosing Spondylitis Disease Activity Score–C-reactive protein; ASQoL = Ankylosing Spondylitis Quality of Life; BASDAI = Bath Ankylosing Spondylitis Disease Activity Index; BASFI = Bath Ankylosing Spondylitis Functional Index; BASMI = Bath Ankylosing Spondylitis Metrology Index; Hedges g = Bias-corrected standardized mean difference (on change scores); CI = Confidence interval.

Outcome	Interval	Comparison (ΔΔ)	n=(10 mg/15 mg)	ΔΔ mean (95% CI)	Hedges g (95% CI)
ASDAS-CRP	Δ 0→12	ΔΔ (10 mg − 15 mg)	57/31	-1.44 (-1.70 to -1.19)	-2.49 (-3.18 to -1.96)
ASDAS-CRP	Δ 0→24	ΔΔ (10 mg − 15 mg)	57/31	-0.80 (-1.19 to -0.41)	-1.00 (-1.57 to -0.52)
ASQoL	Δ 0→12	ΔΔ (10 mg − 15 mg)	57/31	-6.26 (-8.50 to -4.03)	-1.44 (-2.07 to -0.94)
ASQoL	Δ 0→24	ΔΔ (10 mg − 15 mg)	57/31	-4.17 (-6.15 to -2.18)	-1.11 (-1.61 to -0.68)
BASDAI	Δ 0→12	ΔΔ (10 mg − 15 mg)	57/31	-1.45 (-1.88 to -1.01)	-1.40 (-1.89 to -1.00)
BASDAI	Δ 0→24	ΔΔ (10 mg − 15 mg)	57/31	-0.72 (-1.23 to -0.22)	-0.62 (-1.06 to -0.21)
BASFI	Δ 0→12	ΔΔ (10 mg − 15 mg)	57/31	-1.74 (-2.38 to -1.10)	-1.28 (-1.84 to -0.83)
BASFI	Δ 0→24	ΔΔ (10 mg − 15 mg)	57/31	-1.46 (-2.13 to -0.78)	-0.98 (-1.51 to -0.53)
BASMI (linear)	Δ 0→12	ΔΔ (10 mg − 15 mg)	57/31	-0.20 (-0.49 to 0.09)	-0.31 (-0.78 to 0.14)
BASMI (linear)	Δ 0→24	ΔΔ (10 mg − 15 mg)	57/31	-0.09 (-0.40 to 0.23)	-0.14 (-0.61 to 0.33)

Subgroup analyses

Both treatment groups demonstrated substantial and statistically significant improvements across all measured outcomes, with 95% confidence intervals excluding zero, indicating that the changes were unlikely due to chance. From baseline to week 12, the 10 mg group showed a greater reduction in ASDAS-CRP (Δ = −2.58, effect size dz = −4.47) compared to the 15 mg group (Δ = −1.13, dz = −1.98). By week 24, ASDAS-CRP continued to improve, reaching −2.66 in the 10 mg group and −1.86 in the 15 mg group. Similar large improvements were observed in ASQoL, BASDAI, and BASFI scores for both groups, with consistently greater effect sizes in the 10 mg group. BASMI also improved moderately in both groups, again with slightly more pronounced gains in the 10 mg group. Detailed results are presented in the Appendices (Table 5).

By age (≤34 vs >34 years), treatment responses were consistent, with no evidence of a dose-by-age interaction for any outcome (all interaction p-values > 0.38). Both age groups demonstrated large, clinically meaningful improvements within each dose, and the pattern of between-dose differences was consistent with the overall findings (Appendices, Table 6).

No dose-by-sex interactions were observed for ASDAS-CRP, ASQoL, BASDAI, BASFI, or BASMI at either timepoint (all interaction p > 0.46). Within both male and female groups, effect sizes were large and followed the same directional pattern as the overall results (Appendices, Table 7)

When stratified by disease duration (≤7 vs >7 years), no significant dose-by-duration interactions were observed for most outcomes. At week 12, two interactions emerged: a greater 10 mg benefit for BASDAI in participants with longer disease duration (interaction p = 0.0056) and a modest interaction for ASQoL (p = 0.0448). However, both effects diminished by week 24 and were no longer statistically significant (Appendices, Table 8).

By disease duration (≤7 vs >7 years), most outcomes showed no interaction. Two week-12 signals emerged - a stronger 10-mg benefit for BASDAI in those with longer duration (interaction p=0.0056) and a modest interaction for ASQoL (p=0.0448)-both of which attenuated by week 24 (NS) (Appendices, Table 9).

When stratified by baseline ASDAS-CRP severity (<3.5 vs ≥3.5), no evidence of effect modification was observed for any outcome at either time point (all interaction p > 0.51). Both severity groups showed marked improvements within each dose, and the between-dose differences were consistent with the primary analysis (Appendices, Table 10).

**Figure 2:**
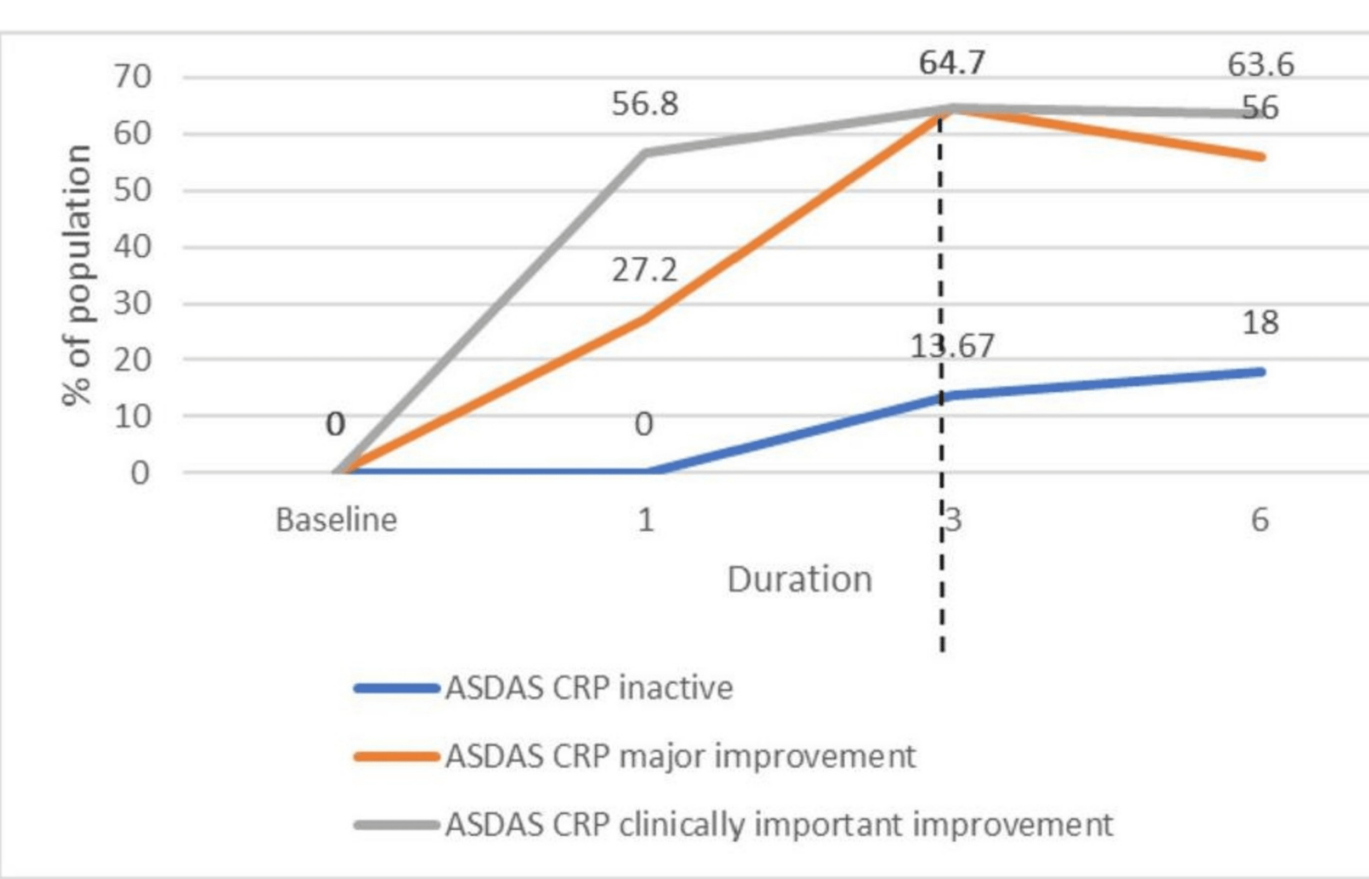
ASDAS-CRP response states over time (CII, MI, ID) in the full evaluable cohort; week 12 is the dose-escalation point (n=88 at weeks 12 and 24). Proportions calculated on the evaluable cohort at each time point. All patients at baseline were enrolled with ASDAS-CRP high/very high disease activity (>2.1). CII = Clinically important improvement; MI = Major improvement; ID = Inactive disease; ASDAS-CRP = Ankylosing Spondylitis Disease Activity Score - C-reactive protein

At week 12 (vertical dashed line, dose-escalation point), 64.8% achieved ASDAS-CRP MI, 83.0% achieved CII, and 13.6% were Inactive Disease (ID). By week 24, MI was 67.0%, CII 90.9%, and ID 20.5%. These percentages are calculated on the full evaluable cohort at each time point (n=88 at weeks 12 and 24) (Figure 2).

**Figure 3:**
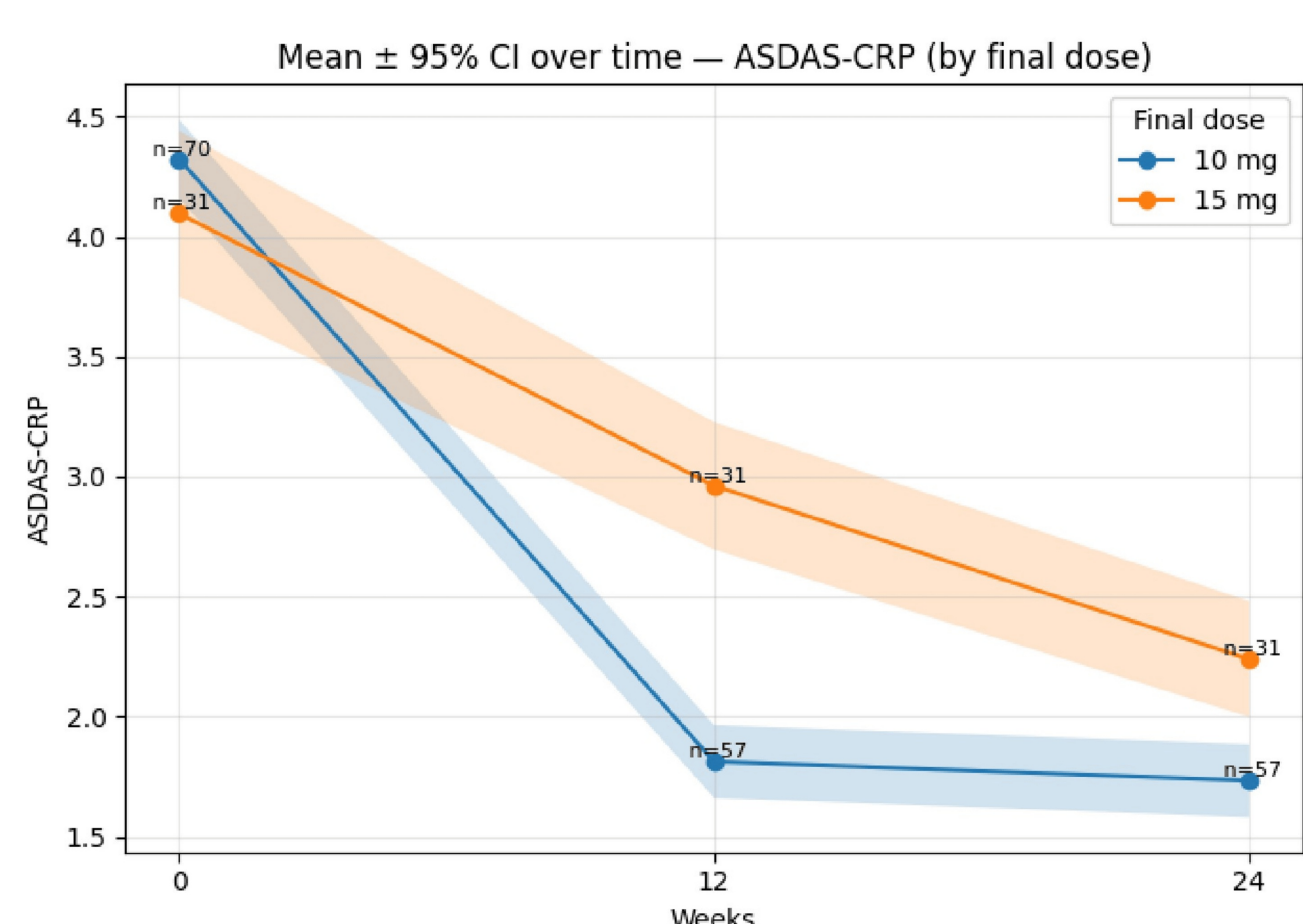
Mean ASDAS-CRP (±95% CI) over time by final dose (10 mg vs 15 mg); baseline n=70/31; weeks 12 & 24 n=57/31. Error bands represent 95% CIs. ASDAS-CRP = Ankylosing Spondylitis Disease Activity Score - C-reactive protein; CI = Confidence interval.

Mean ASDAS-CRP (±95% CI) over time by final dose (10 mg vs 15 mg); baseline n=70/31; weeks 12 and 24 n=57/31 is shown in Figure 3.

**Figure 4:**
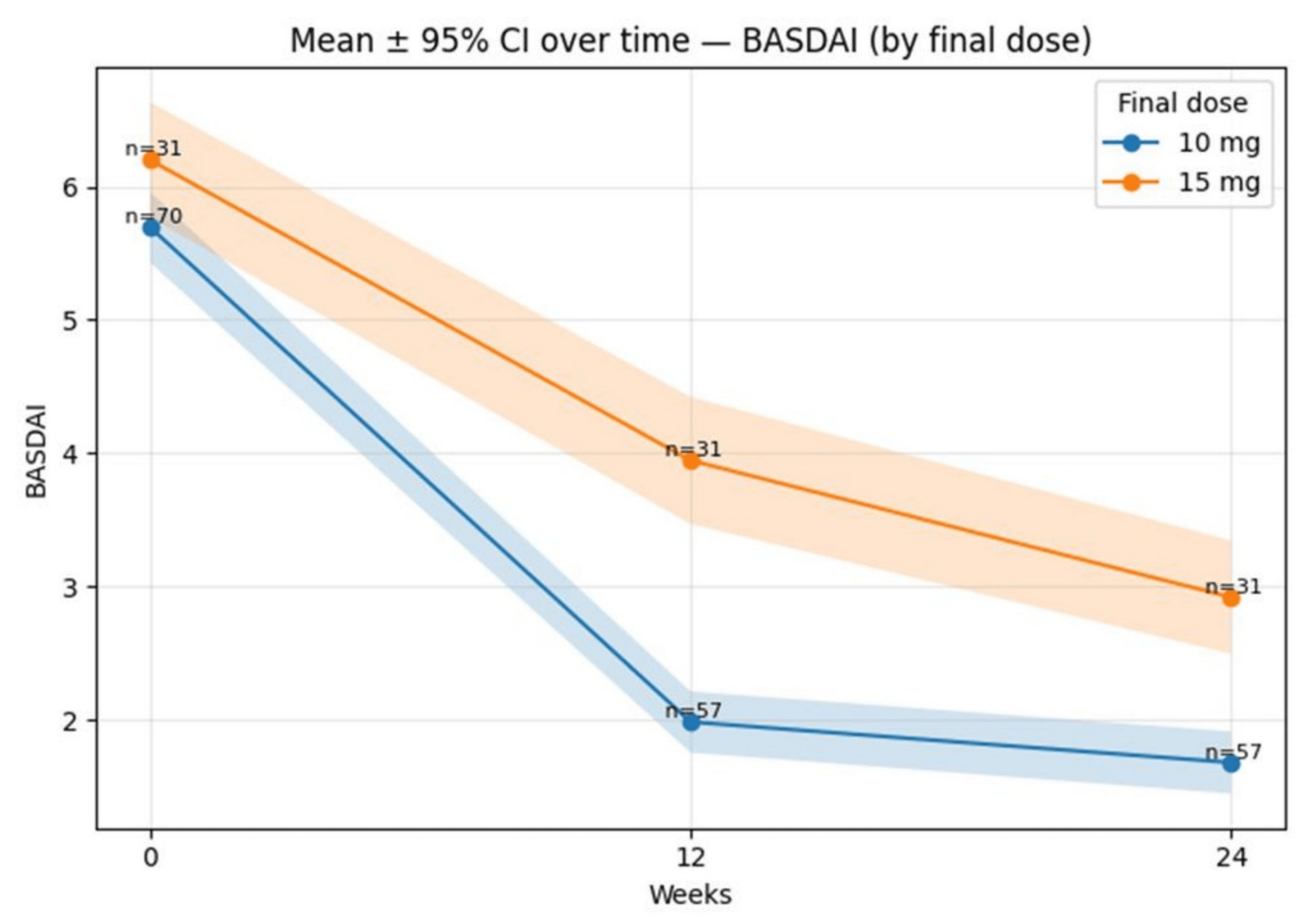
Mean BASDAI (±95% CI) over time by final dose (10 mg vs 15 mg). Error bands represent 95% CIs; BASDAI = Bath Ankylosing Spondylitis Disease Activity Index; CI = Confidence interval.

Mean BASDAI (±95% CI) over time by final dose (10 mg vs 15 mg) is shown in Figure 4.

**Figure 5:**
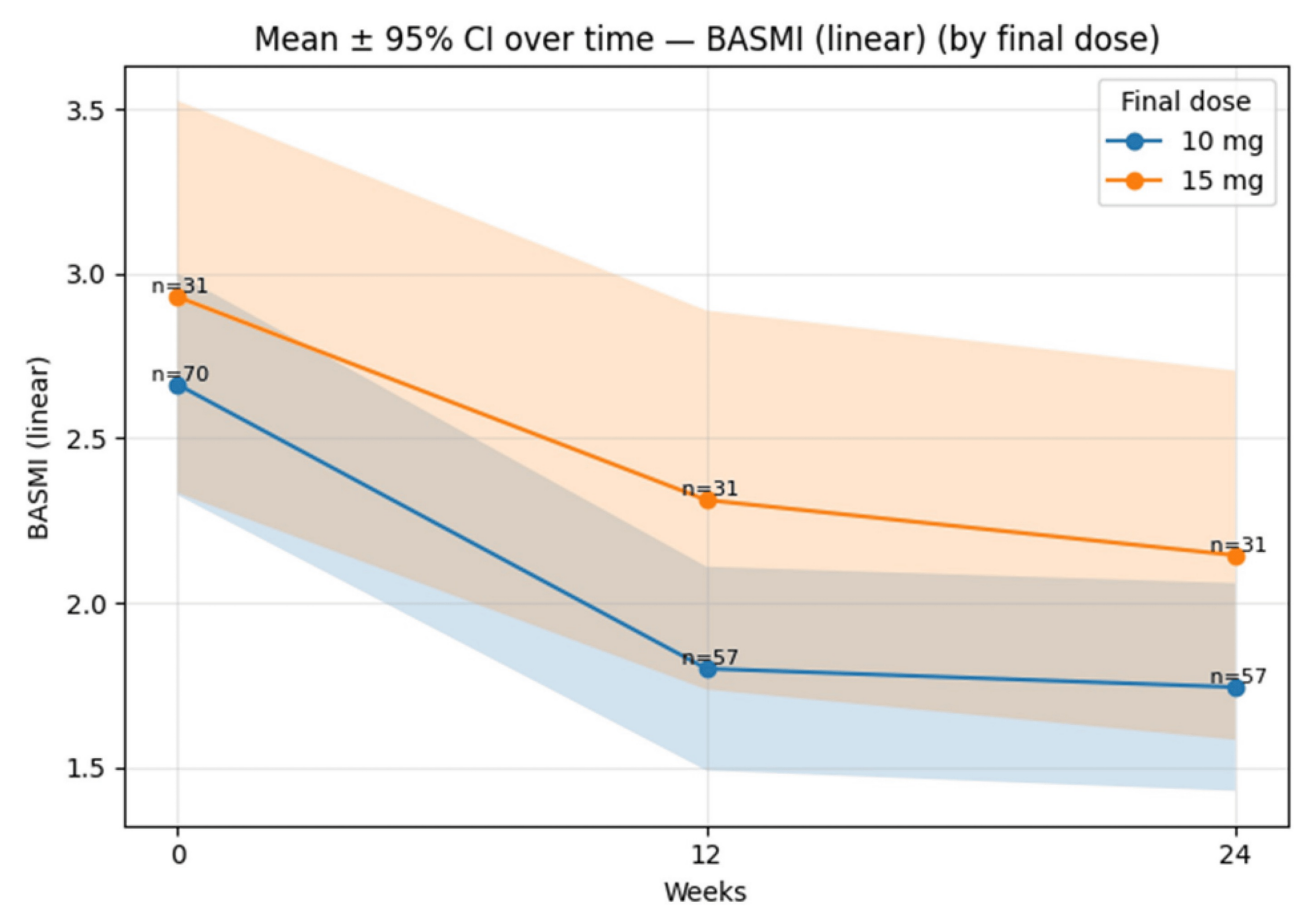
Mean BASMI (linear; ±95% CI) over time by final dose (10 mg vs 15 mg). Error bands represent 95% CIs; BASMI = Bath Ankylosing Spondylitis Metrology Index; CI = Confidence interval.

BASMI declined from ~2.6 (10 mg) and ~2.9 (15 mg) at baseline to ~1.8 and ~2.3 at 12 weeks, and ~1.7 and ~2.1 at 24 weeks, as shown in Figure 5.

**Figure 6:**
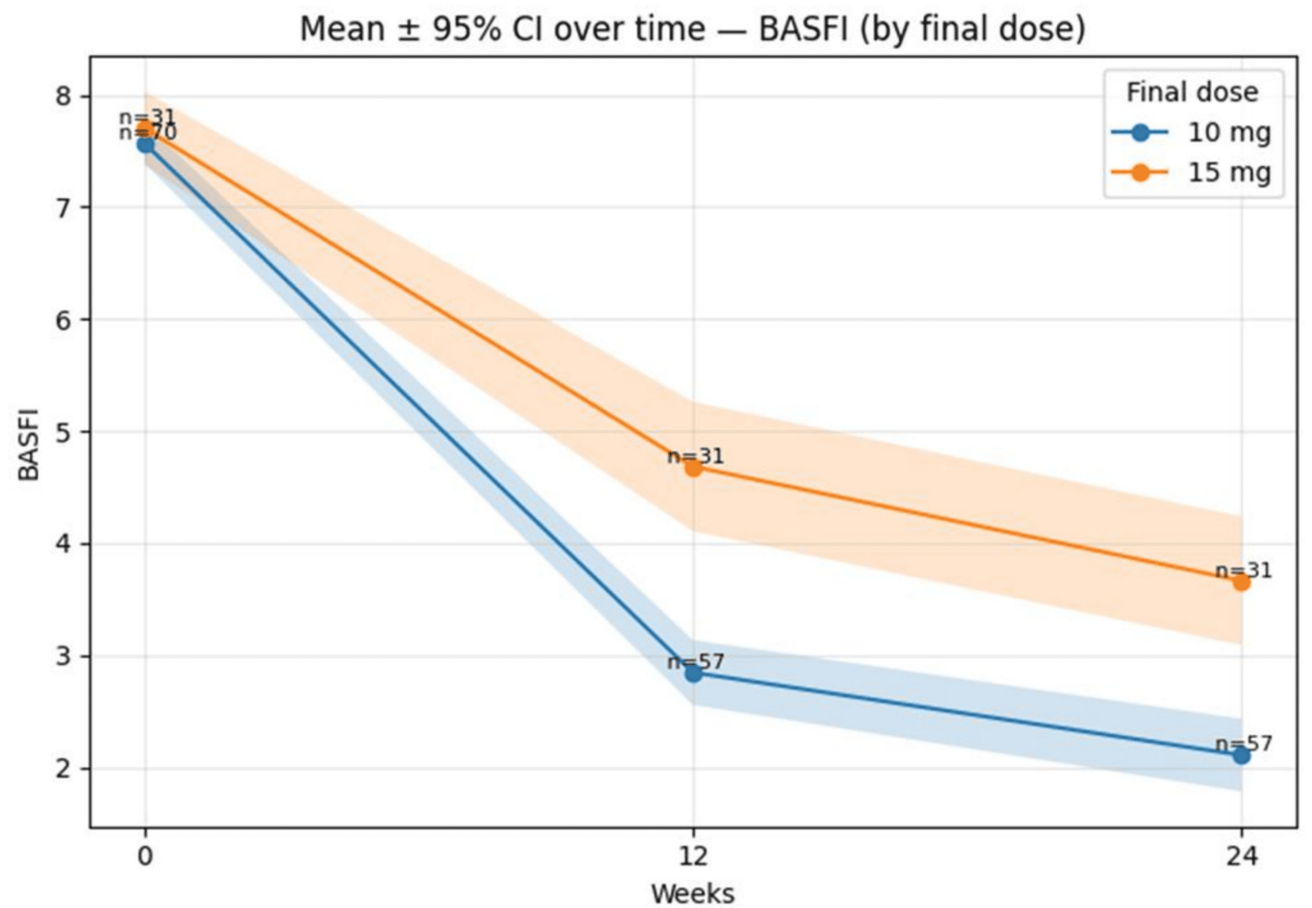
Mean BASFI (±95% CI) over time by final dose (10 mg vs 15 mg). Error bands represent 95% CIs, BASFI = Bath Ankylosing Spondylitis Functional Index; CI = Confidence interval.

Mean BASFI (±95% CI) over time by final dose (10 mg vs 15 mg) is shown in Figure 6

**Figure 7:**
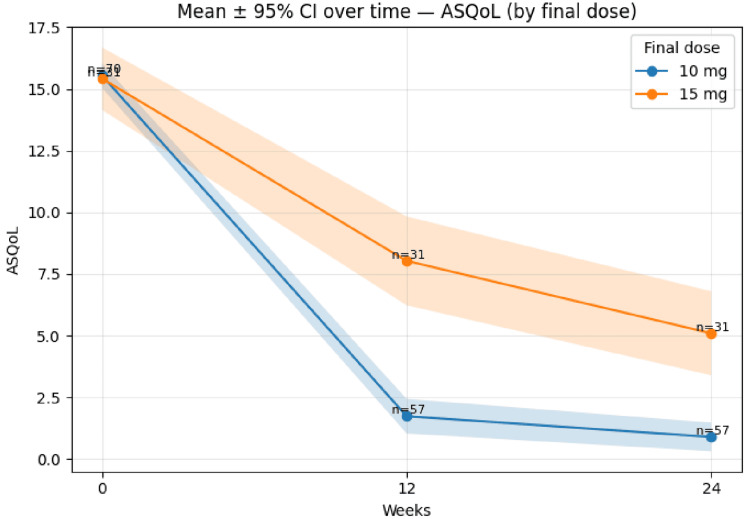
Mean ASQoL (±95% CI) over time by final dose (10 mg vs 15 mg). Error bands represent 95% CIs, ASQoL = Ankylosing Spondylitis Quality of Life; CI = Confidence interval.

ASQoL improved from ~15.3 in both groups at baseline to ~1.5 (10 mg) and ~7.9 (15 mg) at 12 weeks, and ~0.9 and ~5.0 at 24 weeks (Figure 7).

**Figure 8:**
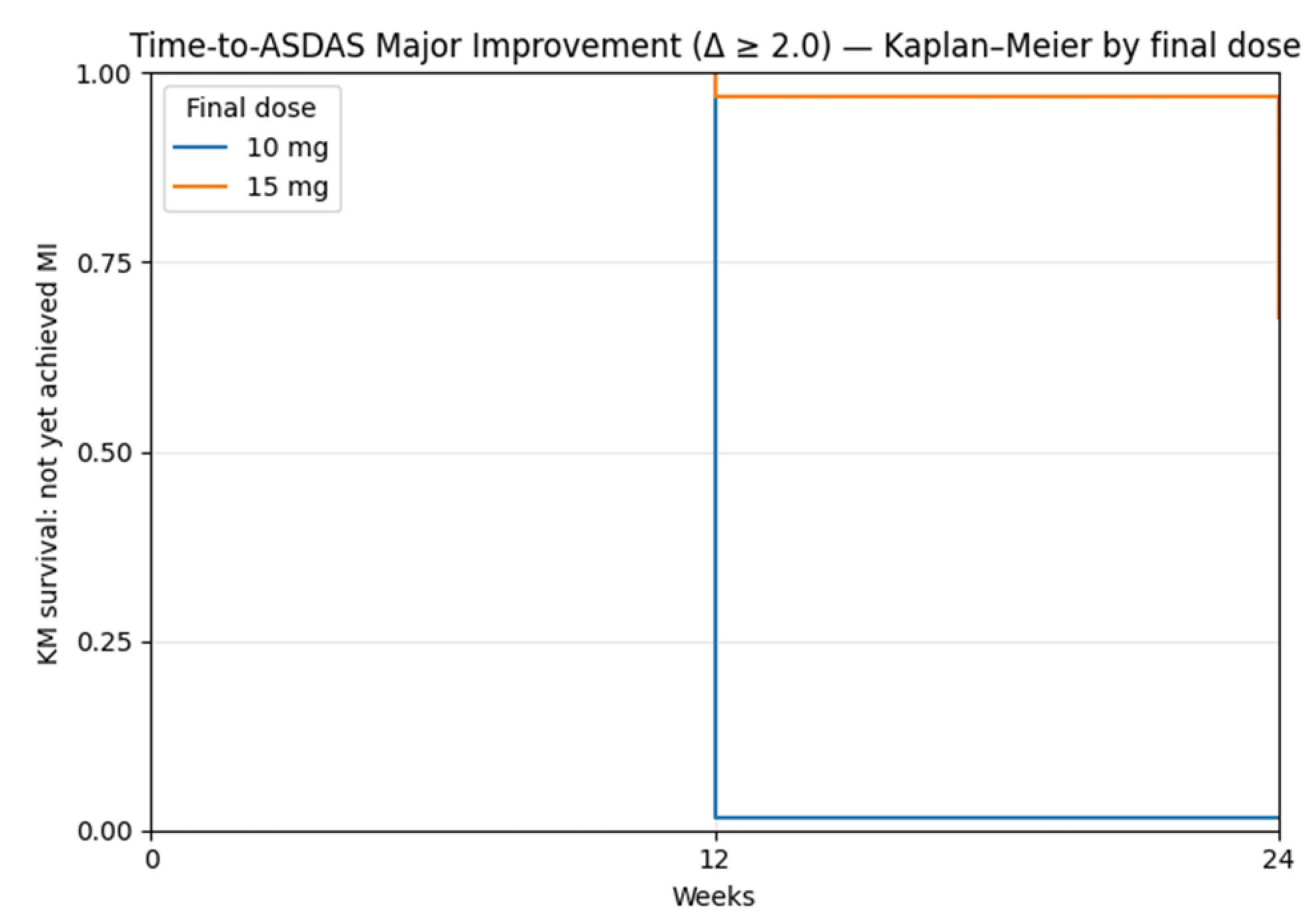
Time to ASDAS major improvement (Δ≥2.0): Kaplan-Meier curves by final dose through week 24. The  patients who stayed on 10 mg reached Major Improvement(MI) by week 12, while ~32% of those on a final dose of 15 mg achieved MI by week 24. Blue Line (10 mg final dose): Stays at 1.0 until week 12, then drops sharply to 0.This means that all patients still on 10 mg achieved MI by week 12.This is expected, as per study design (patients not improving by week 12 likely escalated to 15 mg).Orange Line (15 mg final dose):Stays flat at 1.0 longer and only drops slightly by week 24. Ends at around 0.68, meaning that only about 32% achieved MI by week 24, while 68% had still not achieved MI. Indicates a slower or less frequent response in the 15 mg group. ASDAS = Ankylosing Spondylitis Disease Activity Score; MI = Major improvement; KM = Kaplan–Meier; CI = Confidence interval.

The Kaplan-Meier curves illustrate the probability of not yet achieving Major improvement (MI) over time within each final-dose subgroup, showing that all patients who remained on a final dose of 10 mg had achieved MI by week 12, as indicated by the KM curve dropping to approximately zero, whereas in the final-dose 15 mg group, around 32% of patients had achieved MI by week 24, reflected by the KM estimate remaining at approximately 0.68 (Figure 8).

Across 24 weeks, adverse events were common but mostly comparable between doses. The only significant difference was rhinitis, higher with 15 mg (58.1% vs 31.6%; p=0.016). Fever and pharyngitis were frequent but not different (p=0.408, 0.228). Dry cough trended higher with 15 mg (32.3% vs 15.8%; p=0.073). Serious events were rare and similar: pneumonia, chest pain (ischemic heart disease (IHD)), and raised SGPT (8.8% vs 9.7%; p=1.0). One malignancy occurred in the 10 mg group. No cases of TB, diarrhoea, UTI, leukopenia, or thrombocytopenia were observed. Overall tolerability was comparable between 10 mg and 15 mg (Table 4).

**Table 4:** Adverse effects of patients with tofacitinib 10 mg vs tofacitinib 15 mg at 24 weeks ^*^χ^2^ test UTI = Urinary Tract Infection; Hb = hemoglobin; IHD = ischemic heart disease; SGPT = serum glutamic-pyruvic transaminase

Adverse effects	Tofacitinib 10mg n=57, (%)	Tofacitinib 15mg n=31, (%)	p-value*
Fever	26 (45.6)	17 (54.8)	0.408
Headache	3 (5.3)	4 (12.9)	0.236
Fatigue	4 (7)	2 (6.5)	1
Edema	1 (1.8)	0 (0.0)	1
Rhinitis	18 (31.6)	18 (58.1)	0.016
Pharyngitis	15 (26.3)	12 (38.7)	0.228
Dry cough	9 (15.8)	10 (32.3)	0.073
Pneumonia	2 (3.5)	1 (3.2)	1
Tuberculosis	0 (0.0)	0 (0.0)	NA
Diarrhoea	0 (0.0)	0 (0.0)	NA
Nausea	2 (3.5)	3 (9.7)	0.34
Vomiting	0 (0.0)	1 (3.2)	0.352
Dry mouth	0 (0.0)	1 (3.2)	1
Oral ulcer	1 (1.8)	1 (3.2)	1
Dyspepsia	3 (5.3)	0 (0.0)	0.549
Abdominal pain	3 (5.3)	1 (3.2)	1
Hypertension	0 (0.0)	1 (3.2)	0.352
Chest pain (IHD)	2 (3.5)	4 (12.9)	0.179
Dyslipidemia	13 (22.8)	9 (29.0)	0.519
Paresthesia	1 (1.8)	0 (0.0)	1
Dysuria	0 (0.0)	0 (0.0)	NA
UTI	0 (0.0)	0 (0.0)	NA
Rash	9 (15.8)	5 (16.1)	1
Purpura	1 (1.8)	0 (0.0)	1
Fungal	4 (7.0)	2 (6.5)	1
Herpes Zoster	0 (0.0)	1 (3.2)	0.352
Pruritus	8 (14.0)	3 (9.7)	0.74
Scabies	4 (7.0)	1 (3.2)	0.653
Hyperpigmentation	1 (1.8)	2 (6.5)	0.282
Abscess	1 (1.8)	2 (6.5)	0.282
Raised SGPT	5 (8.8)	3 (9.7)	1
Joint pain	4 (7.0)	4 (12.9)	0.445
Muscle pain	4 (7.0)	1 (3.2)	0.653
Hb drop	4 (7.0)	2 (6.5)	1
Thrombocytopenia	0 (0.0)	0 (0.0)	NA
Leukopenia	0 (0.0)	0 (0.0)	NA
Malignancy	1 (1.8)	0 (0.0)	0.352

## Discussion

In this study, 88 evaluable patients with active axial spondyloarthritis received tofacitinib 10 mg once daily for the initial 12 weeks. At week 12, 64.8% (n=57) achieved ASDAS-CRP MI, 83.0% achieved clinically important improvement, and 13.6% reached inactive disease. Patients meeting MI at week 12 (n=57) continued 10 mg; the remaining 31 patients (35.2%) escalated to 15 mg from week 12 onward. Cross-trial efficacy comparisons (e.g., with phase 3 data) are not made here due to design and population differences [23].

A total of 101 patients were enrolled in the intent-to-treat (ITT) population, with 88 completing both the week 12 and week 24 assessments. At the week 12 dose-escalation point, 64.8% achieved ASDAS-CRP major improvement (MI), 83.0% showed clinically important improvement (CII), and 13.6% reached inactive disease (ID). By week 24, these proportions increased to 67.0%, 90.9%, and 20.5%, respectively (evaluable cohort at each time point: n=88).

Patients who remained on the 10 mg dose (i.e., those who achieved MI by week 12 and did not escalate) demonstrated substantial within-group improvements from baseline to weeks 12 and 24 in key outcomes: ASDAS-CRP decreased by −2.58 and −2.66, BASDAI by −3.70 and −4.00, BASFI by −4.76 and −5.50, and BASMI by −0.82 and −0.87, respectively. 

Among patients who escalated to 15 mg at week 12 (those not achieving MI), improvements from baseline to week 24 were also observed: ASDAS-CRP decreased by −1.86, BASDAI by −3.28, and BASFI by −4.04. Additional gains between weeks 12 and 24 were noted in physical function and quality of life measures, including BASFI (from 4.2 ± 1.5 to 3.4 ± 1.7), B-HAQ-DI (from 0.9 ± 0.6 to 0.7 ± 0.6), SF-36 Physical Component Score (from 37.0 ± 8.7 to 43.9 ± 10.3), and Mental Component Score (from 40.6 ± 11.2 to 47.8 ± 8.1).The magnitude and speed of improvement align with post hoc kinetics seen with JAK inhibitors; in non-responders at 12 weeks, dose escalation offered additional benefit without new safety signals, suggesting a treat-to-target escalation step in constrained settings before switching classes [24-25]. Responders showed rapid clinical improvement as early as 2 weeks, stabilizing by week 4. Those without early response benefited from dose escalation. ASAS 40 response increased gradually, peaking at 24 weeks.

Baseline differences between the week-12 MI (final dose 10 mg) and non-MI (escalation to 15 mg) groups included older age (38.3 ± 9.9 vs 31.5 ± 9.7 years; p=0.003) and higher age at diagnosis (35.0 ± 10.1 vs 28.4 ± 8.5 years; p=0.003) in the escalation group. More participants in the escalation group had education up to Secondary School Certificate (SSC)** **or less (77.5% vs 50.8%; p=0.096, trend) and longer work absence (9.6 ± 16.7 vs 4.8 ± 7.0 months; p=0.129, trend). These characteristics were associated with not meeting MI at week 12 in univariate comparisons, but most were not statistically significant and should not be interpreted as independent predictors.

The safety profile was consistent with previous studies [26-29 ]. Common adverse events included rhinitis, pharyngitis, and dry cough, with no significant differences between doses. Dyslipidemia was noted in 25.7% overall, lower than a recent Bangladeshi study [30], mainly affecting triglycerides and LDL cholesterol. Five patients discontinued due to elevated SGPT within four weeks on 10 mg. One malignancy (breast fibroadenoma) occurred in the 10 mg group. Novel adverse events such as patchy facial hyper/hypopigmentation and new-onset scabies were reported, not previously documented.

Limitations of the study

The study has several limitations, including a single-arm design, non-randomized allocation by response (risk of regression to the mean), a modest sample, attrition, and potential selection and performance biases. Several patient-related factors were identified that may be associated with the failure to meet clinical response criteria and subsequent dose escalation. These include older age, longer disease duration, delayed diagnosis, extended gaps from work due to disease, and lower educational levels. Future research with randomized, blinded designs and comprehensive pharmacokinetic (PK) monitoring is needed to strengthen the evidence base.

## Conclusions

Patients who did not achieve major improvement (MI) on 10 mg by week 12 and increased to 15 mg showed improvements in disease activity and quality of life through week 24. At week 24, 10.2% achieved major improvement (MI) based on a fixed assessment, but Kaplan-Meier analysis shows about 32% achieved Major Improvement by that time. Tofacitinib 15 mg may be a good option for those not responding to 10 mg, but because the study was non-randomized, firm conclusions about its effectiveness cannot be made. Larger and longer studies are needed to confirm these findings and evaluate safety.

## Appendices

**Table 5:** Within-group changes by dose over time across clinical outcomes. Δ = Change from baseline; ASDAS-CRP = Ankylosing Spondylitis Disease Activity Score - C-reactive protein; ASQoL = Ankylosing Spondylitis Quality of Life; BASDAI = Bath Ankylosing Spondylitis Disease Activity Index; BASFI = Bath Ankylosing Spondylitis Functional Index; BASMI = Bath Ankylosing Spondylitis Metrology Index; CI = Confidence interval; Cohen’s dz = Standardized mean change (mean change ÷ SD of change); SRM = Standardized response mean.

Outcome	Interval	Dose mg/mg	n=number	Mean Change (95% CI)	Cohen’s dz	SRM
ASDAS-CRP	Δ 0→12	10 / 15	57 / 31	-2.58 (-2.73, -2.43) / -1.13 (-1.34, -0.92)	-4.47 / -1.98	-4.47 / -1.98
	Δ 0→24	10 / 15	57 / 31	-2.66 / -1.86	-3.84 / -1.95	-3.84 / -1.95
ASQoL	Δ 0→12	10 / 15	57 / 31	-13.65 / -7.39	-4.03 / -1.31	-4.03 / -1.31
	Δ 0→24	10 / 15	57 / 31	-14.49 / -10.32	-5.26 / -2.03	-5.26 / -2.03
BASDAI	Δ 0→12	10 / 15	57 / 31	-3.70 / -2.25	-3.44 / -2.44	-3.44 / -2.44
	Δ 0→24	10 / 15	57 / 31	-4.00 / -3.28	-3.34 / -3.03	-3.34 / -3.03
BASFI	Δ 0→12	10 / 15	57 / 31	-4.76 / -3.02	-3.83 / -1.99	-3.83 / -1.99
	Δ 0→24	10 / 15	57 / 31	-5.50 / -4.04	-3.89 / -2.58	-3.89 / -2.58
BASMI (linear)	Δ 0→12	10 / 15	57 / 31	-0.82 / -0.62	-1.31 / -0.94	-1.31 / -0.94
	Δ 0→24	10 / 15	57 / 31	-0.87 / -0.78	-1.49 / -1.04	-1.49 / -1.04

**Table 6:** Subgroup analysis by age (≤34 vs >34 years): within-dose mean changes and dose× subgroup interactions at weeks 12 and 24 (10 mg vs 15 mg). Δ = Change from baseline; β = Interaction coefficient (dose × subgroup); ASDAS-CRP = Ankylosing Spondylitis Disease Activity Score - C-reactive protein; ASQoL = Ankylosing Spondylitis Quality of Life; BASDAI = Bath Ankylosing Spondylitis Disease Activity Index; BASFI = Bath Ankylosing Spondylitis Functional Index; BASMI = Bath Ankylosing Spondylitis Metrology Index; CI = Confidence interval; Cohen’s dz = Standardized mean change; p = p-value.

Outcome	Interval /week)	Age Subgroup	Dose/mg	n	Δ Mean (95% CI)	Cohen’s dₓ	Interaction β (Dose × Subgroup) [95% CI]	P-value (Interaction)
ASDAS-CRP	0–12	>34 years	10	23	–2.49 (–2.71 to –2.27)	–4.87	0.01 (–0.40 to 0.41)	0.9775
		-	15	19	–1.01 (–1.28 to –0.74)	–1.82	-	-
		≤34 years	10	34	–2.64 (–2.85 to –2.42)	–4.28	-	--
		-	15	12	–1.33 (–1.69 to –0.97)	–2.35	-	
	0–24	>34 years	10	23	–2.45 (–2.70 to –2.19)	–4.19	–0.01 (–0.48 to 0.47)	0.9765
		-	15	19	–1.62 (–2.05 to –1.18)	–1.80	-	-
		≤34 years	10	34	–2.80 (–3.06 to –2.55)	–3.83	-	-
		-	15	12	–2.23 (–2.83 to –1.63)	–2.37	-	-
ASQoL	0–12	>34 years	10	23	–12.35 (–14.05 to –10.64)	–3.14	–0.59 (–3.81 to 2.62)	0.7149
		-	15	19	–7.05 (–9.48 to –4.63)	–1.40	-	-
		≤34 years	10	34	–14.53 (–15.46 to –13.60)	–5.45	-	-
		-	15	12	–7.92 (–12.17 to –3.66)	–1.18	-	--
	0–24	>34 years	10	23	–13.43 (–14.82 to –12.05)	–4.19	–0.07 (–2.91 to 2.78)	0.9629
		-	15	19	–9.89 (–12.15 to –7.63)	–2.11	-	-
		≤34 years	10	34	–15.21 (–15.96 to –14.45)	–7.00		-
		-	15	12	–11.00 (–14.67 to –7.33)	–1.90	--	-
BASDAI	0–12	>34 years	10	23	–3.52 (–3.98 to –3.06)	–3.29	–0.26 (–1.05 to 0.52)	0.5055
BASFI	-	-	15	19	–2.32 (–2.81 to –1.82)	–2.24	-	-
	-	≤34 years	10	34	–3.82 (–4.20 to –3.45)	–3.55	-	-
	-	--	15	12	–2.16 (–2.63 to –1.69)	–2.91	-	-
	0–24	>34 years	10	23	–3.67 (–4.19 to –3.15)	–3.05	–0.34 (–1.13 to 0.44)	0.3859
	-	-	15	19	–3.30 (–3.92 to –2.68)	–2.56		-
	-	≤34 years	10	34	–4.23 (–4.63 to –3.82)	–3.64	-	-
	-	-	15	12	–3.25 (–3.69 to –2.80)	–4.63	-	-
	0–12	>34 years	10	23	–4.44 (–4.97 to –3.91)	–3.62	–0.01 (–1.14 to 1.11)	0.9803
	-	--	15	19	–2.81 (–3.60 to –2.01)	–1.71		-
	-	≤34 years	10	34	–4.98 (–5.40 to –4.55)	–4.07	-	-
	-	-	15	12	–3.36 (–4.17 to –2.54)	–2.62	-	
	0–24	>34 years	10	23	–5.08 (–5.78 to –4.38)	–3.16	0.31 (–0.87 to 1.49)	0.6021
	-	-	15	19	–3.64 (–4.44 to –2.84)	–2.19	-	-
	-	≤34 years	10	34	–5.78 (–6.20 to –5.36)	–4.79	-	-
	-	-	15	12	–4.67 (–5.44 to –3.91)	–3.90	-	-
BASMI (Linear)	0–12	>34 years	10	23	–0.87 (–1.11 to –0.63)	–1.56	0.16 (–0.37 to 0.70)	0.5428
	-	-	15	19	–0.56 (–0.83 to –0.28)	–0.98	-	-
	-	≤34 years	10	34	–0.78 (–1.02 to –0.55)	–1.17	--	-
	-	-	15	12	–0.52 (–0.84 to –0.20)	–0.93	-	-

**Table 7:** Subgroup analysis by age (≤34 vs >34 years): within-dose mean changes and dose×subgroup interactions at weeks 12 and 24 (10 mg vs 15 mg). Δ = Change from baseline; β = Interaction coefficient (dose × subgroup); ASDAS-CRP = Ankylosing Spondylitis Disease Activity Score - C-reactive protein; ASQoL = Ankylosing Spondylitis Quality of Life; BASDAI = Bath Ankylosing Spondylitis Disease Activity Index; BASFI = Bath Ankylosing Spondylitis Functional Index; BASMI = Bath Ankylosing Spondylitis Metrology Index; CI = Confidence interval; Cohen’s dz = Standardized mean change; p = p-value.

Outcome	Interval	Level (Median)	Dose/mg	n	Δ mean (95% CI)	Cohen dz	Interaction β (dose×subgroup) [95% CI]	p (interaction)
ASDAS-CRP	Δ 0→12 Δ 0→12 Δ 0→12 Δ 0→12	>(34.0y)	10	23	-2.49 (-2.71 to -2.27)	-4.87	0.01 (-0.40 to 0.41)	0.9775
		> 34.0y)	15	19	-1.01 (-1.28 to -0.74)	-1.82	-	-
		≤ 34.0y)	10	34	-2.64 (-2.85 to -2.42)	-4.28	-	-
		≤ 34.0y)	15	12	-1.33 (-1.69 to -0.97)	-2.35	-	-
	Δ 0→24	> 34.0y)	10	23	-2.45 (-2.70 to -2.19)	-4.19	-0.01 (-0.48 to 0.47)	0.9765
		>(34.0y)	15	19	-1.62 (-2.05 to -1.18)	-1.8	-	-
		≤ 34.0y)	10	34	-2.80 (-3.06 to -2.55)	-3.83	-	--
		≤ 34.0y)	15	12	-2.23 (-2.83 to -1.63)	-2.37	-	-
ASQoL	Δ 0→12	> 34.0y)	10	23	-12.35 (-14.05 to -10.64)	-3.14	-0.59 (-3.81 to 2.62)	0.7149
		> 34.0y)	15	19	-7.05 (-9.48 to -4.63)	-1.4	--	-
		≤ 34.0y)	10	34	-14.53 (-15.46 to -13.60)	-5.45	-	-
		≤ 34.0y)	15	12	-7.92 (-12.17 to -3.66)	-1.18		
	Δ 0→24	>(34.0y)	10	23	-13.43 (-14.82 to -12.05)	-4.19	-0.07 (-2.91 to 2.78)	0.9629
		>(34.0y)	15	19	-9.89 (-12.15 to -7.63)	-2.11	-	--
		≤ (34.0y)	10	34	-15.21 (-15.96 to -14.45)	-7	-	-
		≤ 34.0y)	15	12	-11.00 (-14.67 to -7.33)	-1.9	-	-
BASDAI	Δ 0→12	> 34.0y)	10	23	-3.52 (-3.98 to -3.06)	-3.29	-0.26 (-1.05 to 0.52)	0.5055
		> 34.0y)	15	19	-2.32 (-2.81 to -1.82)	-2.24	--	-
		≤ 34.0y)	10	34	-3.82 (-4.20 to -3.45)	-3.55	-	-
		≤ 34.0y)	15	12	-2.16 (-2.63 to -1.69)	-2.91	-	-
	Δ 0→24	> 34.0y)	10	23	-3.67 (-4.19 to -3.15)	-3.05	-0.34 (-1.13 to 0.44)	0.3859
		>(34.0y)	15	19	-3.30 (-3.92 to -2.68)	-2.56	--	-
		≤ 34.0y)	10	34	-4.23 (-4.63 to -3.82)	-3.64	--	-
		≤ 34.0y)	15	12	-3.25 (-3.69 to -2.80)	-4.63	--	-
BASFI	Δ 0→12	> 34.0y)	10	23	-4.44 (-4.97 to -3.91)	-3.62	-0.01 (-1.14 to 1.11)	0.9803
		> 34.0y)	15	19	-2.81 (-3.60 to -2.01)	-1.71	-	-
		≤ 34.0y)	10	34	-4.98 (-5.40 to -4.55)	-4.07	-	-
		≤ 34.0y)	15	12	-3.36 (-4.17 to -2.54)	-2.62	-	-
	Δ 0→24	> 34.0y)	10	23	-5.08 (-5.78 to -4.38)	-3.16	0.31 (-0.87 to 1.49)	0.6021
		>(34.0y)	15	19	-3.64 (-4.44 to -2.84)	-2.19	--	-
		≤ 34.0y)	10	34	-5.78 (-6.20 to -5.36)	-4.79	-	-
		≤ 34.0y)	15	12	-4.67 (-5.44 to -3.91)	-3.9	-	-
BASMI (linear)	Δ 0→12	> 34.0y)		23	-0.87 (-1.11 to -0.63)	-1.56	0.16 (-0.37 to 0.70)	0.5428
		>(34.0y)	15	19	-0.56 (-0.83 to -0.28)	-0.98	-	-
		≤ 34.0y)	10	34	-0.78 (-1.02 to -0.55)	-1.17	--	-
		≤(34.0y)	15	12	-0.71 (-1.21 to -0.21)	-0.9	-	-
	Δ 0→24	> 34.0y)	10	23	-0.86 (-1.08 to -0.64)	-1.69	-0.06 (-0.61 to 0.48)	0.8255
		>(34.0y)	15	19	-0.77 (-1.11 to -0.42)	-1.07	-	-
		≤ 34.0y)	10	34	-0.88 (-1.11 to -0.66)	-1.37	-	-
		≤(34.0y)	15	12	-0.81 (-1.35 to -0.27)	-0.96	-	-

**Table 8:** Subgroup analysis by sex (female vs male): within-dose mean changes and dose×subgroup interactions at weeks 12 and 24 (10 mg vs 15 mg Δ = Change from baseline; β = Interaction coefficient (dose × subgroup); ASDAS-CRP = Ankylosing Spondylitis Disease Activity Score - C-reactive protein; ASQoL = Ankylosing Spondylitis Quality of Life; BASDAI = Bath Ankylosing Spondylitis Disease Activity Index; BASFI = Bath Ankylosing Spondylitis Functional Index; BASMI = Bath Ankylosing Spondylitis Metrology Index; CI = Confidence interval; Cohen’s dz = Standardized mean change; p = p-value.

Outcome	Interval	Sex	Dose/ mg	n	Δ Mean (95% CI)	Cohen’s dₓ	Interaction β (Dose × Sex) [95% CI]	p (interaction)
ASDAS-CRP	Δ 0→12	Female	10	17	–2.40 (–2.72 to –2.08)	–3.82	–0.10 (–0.52 to 0.32)	0.6501
	-		15	13	–1.08 (–1.37 to –0.80)	–2.30		
	-	Male	10	40	–2.65 (–2.83 to –2.48)	–4.88		
	-		15	18	–1.17 (–1.49 to –0.85)	–1.81		
	Δ 0→24	Female	10	17	–2.34 (–2.66 to –2.01)	–3.68	0.11 (–0.39 to 0.61)	0.6643
	-		15	13	–1.59 (–2.08 to –1.10)	–1.97		
	-	Male	10	40	–2.79 (–3.01 to –2.58)	–4.12		
	-		15	18	–2.05 (–2.55 to –1.54)	–2.01		
ASQoL	Δ 0→12	Female	10	17	–13.24 (–14.83 to –11.65)	–4.28	1.04 (–2.32 to 4.41)	0.54
	-		15	13	–7.00 (–10.02 to –3.98)	–1.40		
	--	Male	10	40	–13.82 (–14.95 to –12.70)	–3.93		
	--		15	18	–7.67 (–10.74 to –4.59)	–1.24		
	Δ 0→24	Female	10	17	–13.47 (–15.13 to –11.81)	–4.18	–0.65 (–3.61 to 2.32)	0.6656
	-		15	13	–10.31 (–13.09 to –7.52)	–2.24		
		Male	10	40	–14.93 (–15.71 to –14.14)	–6.11		
	-		15	18	–10.33 (–13.08 to –7.59)	–1.87		
BASDAI	Δ 0→12	Female	10	17	–3.67 (–4.27 to –3.07)	–3.16	–0.22 (–1.02 to 0.59)	0.5979
	-		15	13	–2.44 (–3.04 to –1.83)	–2.44		
	-	Male	10	40	–3.71 (–4.05 to –3.38)	–3.53		
	-		15	18	–2.12 (–2.55 to –1.69)	–2.45		
	Δ 0→24	Female	10	17	–3.93 (–4.61 to –3.25)	–2.96	0.19 (–0.64 to 1.01)	0.6534
	-		15	13	–3.23 (–3.91 to –2.55)	–2.87		
	-	Male	10	40	–4.04 (–4.41 to –3.67)	–3.49		
	-		15	18	–3.32 (–3.86 to –2.78)	–3.06		
BASFI	Δ 0→12	Female	10	17	–4.13 (–4.79 to –3.47)	–3.23	–0.09 (–1.20 to 1.02)	0.8715
	-		15	13	–2.58 (–3.69 to –1.46)	–1.40		
	-	Male	10	40	–5.03 (–5.39 to –4.66)	–4.41		
	-		15	18	–3.34 (–3.92 to –2.75)	–2.84		
	Δ 0→24	Female	10	17	–4.84 (–5.70 to –3.97)	–2.88	–0.01 (–1.20 to 1.18)	0.9824
			15	13	–3.53 (–4.69 to –2.37)	–1.83		
	-	Male	10	40	–5.78 (–6.16 to –5.39)	–4.82		
	-		15	18	–4.41 (–4.98 to –3.83)	–3.78		
BASMI (Linear)	Δ 0→12	Female	10	17	–0.75 (–1.03 to –0.48)	–1.42	0.20 (–0.34 to 0.74)	0.4671
	-		15	13	–0.43 (–0.69 to –0.18)	–1.02		
	-	Male	10	40	–0.84 (–1.06 to –0.63)	–1.27		
	-		15	18	–0.75 (–1.13 to –0.37)	–0.99		
	Δ 0→24	Female	10	17	–0.82 (–1.07 to –0.58)	–1.74	0.19 (–0.37 to ...)	

**Table 9:** Table S4: Subgroup analysis by disease duration (≤7 vs >7 years): within-dose mean changes and dose×subgroup interactions at weeks 12 and 24 (10 mg vs 15 mg). Δ = Change from baseline; β = Interaction coefficient (dose × subgroup); ASDAS-CRP = Ankylosing Spondylitis Disease Activity Score - C-reactive protein; ASQoL = Ankylosing Spondylitis Quality of Life; BASDAI = Bath Ankylosing Spondylitis Disease Activity Index; BASFI = Bath Ankylosing Spondylitis Functional Index; BASMI = Bath Ankylosing Spondylitis Metrology Index; CI = Confidence interval; Cohen’s dz = Standardized mean change; p = p-value.

Outcome	Interval	Level/median	Dose	n	Δ mean (95% CI)	Cohen dz	Interaction β (dose×subgroup) [95% CI]	p (interaction)
	Δ 0→12	> (7.00)	10 mg	24	-2.52 (-2.77 to -2.28)	-4.31	-0.39 (-0.81 to 0.02)	0.0589
		> (7.00)	15 mg	19	-1.19 (-1.35 to -1.03)	-3.55	--	-
		≤(7.00)	10 mg	33	-2.62 (-2.82 to -2.41)	-4.55	-	-
		≤ (7.00)	15 mg	12	-1.05 (-1.58 to -0.52)	-1.26	--	-
	Δ 0→24	> (7.00)	10 mg	24	-2.52 (-2.83 to -2.20)	-3.42	-0.41 (-0.91 to 0.10)	0.1129
		> (7.00)	15 mg	19	-1.82 (-2.23 to -1.40)	-2.12	-	-
		≤ (7.00)	10 mg	33	-2.76 (-2.99 to -2.53)	-4.24	-	-
		≤ (7.00)	15 mg	12	-1.92 (-2.63 to -1.21)	-1.71	-	-
ASQoL	Δ 0→12	> (7.00)	10 mg	24	-13.71 (-15.06 to -12.36)	-4.29	-3.30 (-6.52 to -0.08)	0.0448
		> (7.00)	15 mg	19	-9.05 (-11.62 to -6.48)	-1.7	-	--
		≤ (7.00)	10 mg	33	-13.61 (-14.87 to -12.34)	-3.82	-	-
		≤ (7.00)	15 mg	12	-4.75 (-8.10 to -1.40)	-0.9	-	--
	Δ 0→24	> (7.00)	10 m	24	-14.46 (-15.64 to -13.28)	-5.17	-0.75 (-3.69 to 2.19)	0.6112
		> (7.00)	15	19	-10.89 (-13.07 to -8.72)	-2.42	-	-
		≤ (7.00)	10	33	-14.52 (-15.49 to -13.54)	-5.25	-	-
		≤ (7.00)	15	12	-9.42 (-13.20 to -5.63)	-1.58	-	-
BASDAI	Δ 0→12	> (7.00)	10	24	-3.81 (-4.32 to -3.30)	-3.14	-1.07 (-1.82 to -0.32)	0.0056
		> (7.00)	15	19	-2.59 (-3.01 to -2.17)	-2.96	--	-
		≤ (7.00)	10	33	-3.62 (-3.97 to -3.28)	-3.73	-	-
		≤ (7.00)	15	12	-1.72 (-2.19 to -1.25)	-2.31	-	-
	Δ 0→24	> (7.00)	10	24	-4.06 (-4.64 to -3.49)	-2.98	-0.76 (-1.58 to 0.07)	0.0712
		> (7.00)	15	19	-3.42 (-4.00 to -2.84)	-2.82	-	-
		≤ (7.00)	10	33	-3.96 (-4.34 to -3.58)	-3.65	---	-
		≤ (7.00)	15	12	-3.06 (-3.60 to -2.52)	-3.6	--	
BASFI	Δ 0→12	> (7.00)	10	24	-4.62 (-5.16 to -4.09)	-3.64	-0.94 (-2.07 to 0.19)	0.1023
		> (7.00)	15	19	-3.21 (-3.98 to -2.43)	-1.99	-	-
		≤ (7.00)	10	33	-4.86 (-5.29 to -4.42)	-3.94	-	-
		≤ (7.00)	15	12	-2.72 (-3.59 to -1.86)	-2	-	-
	Δ 0→24	> (7.00)	10	24	-5.14 (-5.78 to -4.50)	-3.38	-0.85 (-2.05 to 0.36)	0.1671
		> (7.00)	15	19	-4.02 (-4.76 to -3.28)	-2.61	--	-
		≤ (7.00)	10	33	-5.76 (-6.21 to -5.30)	-4.47	-	-
		≤ (7.00)	15	12	-4.07 (-5.13 to -3.01)	-2.44	--	-
BASMI (linear)	Δ 0→12	> (7.00)	10	24	-0.80 (-0.99 to -0.61)	-1.77	-0.19 (-0.74 to 0.35)	0.4812
		> (7.00)	15	19	-0.76 (-1.14 to -0.38)	-0.97	--	--
		≤ (7.00)	10	33	-0.83 (-1.09 to -0.57)	-1.14	-	-
		≤ (7.00)	15	12	-0.38 (-0.52 to -0.25)	-1.77	-	-
	Δ 0→24	> (7.00)	10	24	-0.80 (-0.98 to -0.62)	-1.9	-0.34 (-0.89 to 0.21)	0.2263
		> (7.00)	15	19	-0.95 (-1.39 to -0.52)	-1.05	-	-
		≤ (7.00)	10	33	-0.93 (-1.17 to -0.68)	-1.35	-	-
		≤ (7.00)	15	12	-0.52 (-0.70 to -0.33)	-1.79	-	-

**Table 10:** Subgroup analysis by baseline ASDAS-CRP severity (<3.5 vs ≥3.5): within-dose mean changes and dose×subgroup interactions at weeks 12 and 24 (10 mg vs 15 mg). Δ = Change from baseline; β = Interaction coefficient (dose × subgroup); ASDAS-CRP = Ankylosing Spondylitis Disease Activity Score - C-reactive protein; ASQoL = Ankylosing Spondylitis Quality of Life; BASDAI = Bath Ankylosing Spondylitis Disease Activity Index; BASFI = Bath Ankylosing Spondylitis Functional Index; BASMI = Bath Ankylosing Spondylitis Metrology Index; CI = Confidence interval; Cohen’s dz = Standardized mean change; p = p-value.

Outcome	Interval	Level	Dose/mg	n	Δ mean (95% CI)	Cohen dz	Interaction β (dose× subgroup) [95% CI]	p (interaction)
ASDAS-CRP	Δ 0→12	<3.5	10	5	-2.08 (-2.13 to -2.02)	-45.52	0.15 (-0.41 to 0.71)	0.5942
		<3.5	15	9	-0.77 (-1.04 to -0.50)	-2.22	-	-
		≥3.5	10	52	-2.63 (-2.79 to -2.46)	-4.52	-	-
		≥3.5	15	22	-1.28 (-1.54 to -1.02)	-2.19	-	-
	Δ 0→24	<3.5	10	5	-2.05 (-2.27 to -1.83)	-11.6	-0.16 (-0.86 to 0.53)	0.6424
		<3.5	15	9	-1.14 (-1.51 to -0.77)	-2.37	-	-
		≥3.5	10	52	-2.72 (-2.91 to -2.52)	-3.9	-	
		≥3.5	15	22	-2.15 (-2.57 to -1.73)	-2.27	-	--
ASQoL	Δ 0→12	<3.5	10	5	-13.60 (-18.91 to -8.29)	-3.18	1.23 (-3.16 to 5.61)	0.5793
		<3.5	15	9	-8.33 (-11.64 to -5.03)	-1.94	-	--
		≥3.5	10	52	-13.65 (-14.58 to -12.72)	-4.09	-	--
		≥3.5	15	22	-7.00 (-9.73 to -4.27)	-1.14	-	-
	Δ 0→24	<3.5	10	5	-14.40 (-19.10 to -9.70)	-3.81	0.45 (-3.46 to 4.37)	0.8181
		<3.5	15	9	-10.67 (-13.79 to -7.54)	-2.63	-	-
		≥3.5	10	52	-14.50 (-15.25 to -13.75)	-5.41	-	--
		≥3.5	15	22	-10.18 (-12.63 to -7.74)	-1.85	-	-
BASDAI	Δ 0→12	<3.5	10	5	-3.37 (-4.77 to -1.97)	-2.99	0.35 (-0.71 to 1.41)	0.5155
		<3.5	15	9	-2.16 (-2.85 to -1.47)	-2.42	-	-
		≥3.5	10	52	-3.73 (-4.03 to -3.43)	-3.47	-	-
		≥3.5	15	22	-2.29 (-2.72 to -1.87)	-2.41	-	-
	Δ 0→24	<3.5	10	5	-3.51 (-5.19 to -1.83)	-2.6	0.17 (-0.95 to 1.29)	0.7646
		<3.5	15	9	-2.91 (-3.69 to -2.13)	-2.86	-	-
		≥3.5	10	52	-4.05 (-4.38 to -3.72)	-3.41	--	-
		≥3.5	15	22	-3.43 (-3.92 to -2.95)	-3.13	-	-
BASFI	Δ 0→12	<3.5	10	5	-4.39 (-5.39 to -3.38)	-5.41	0.27 (-1.26 to 1.80)	0.7274
		<3.5	15	9	-2.84 (-4.39 to -1.30)	-1.42	-	-
		≥3.5	10	52	-4.79 (-5.15 to -4.44)	-3.76	-	-
		≥3.5	15	22	-3.09 (-3.67 to -2.51)	-2.36	-	-
	Δ 0→24	<3.5	10	5	-5.23 (-5.81 to -4.65)	-11.18	-0.34 (-1.99 to 1.31)	0.6805
		<3.5	15	9	-3.50 (-5.12 to -1.88)	-1.66	-	--
		≥3.5	10	52	-5.52 (-5.93 to -5.11)	-3.75	-	-
		≥3.5	15	22	-4.26 (-4.82 to -3.70)	-3.35	-	-
BASMI (linear)	Δ 0→12	<3.5	10	5	-0.56 (-1.29 to 0.17)	-0.95	0.16 (-0.56 to 0.87)	0.6586
		<3.5	15	9	-0.58 (-1.14 to -0.01)	-0.78	-	-
		≥3.5	10	52	-0.84 (-1.02 to -0.67)	-1.34	-	-
		≥3.5	15	22	-0.63 (-0.91 to -0.35)	-1	-	-
	Δ 0→24	<3.5	10	5	-0.56 (-1.29 to 0.17)	-0.95	0.17 (-0.56 to 0.90)	0.6438
		<3.5	15	9	-0.71 (-1.39 to -0.04)	-0.81	-	-
		≥3.5	10	52	-0.90 (-1.07 to -0.74)	-1.55	-	-
		≥3.5	15	22	-0.81 (-1.13 to -0.49)	-1.13	-	-

## References

[1] Braun J, van den Berg R, Baraliakos X (2011). 2010 update of the ASAS/EULAR recommendations for the management of ankylosing spondylitis. Ann Rheum Dis.

[2] Misra DP, Sharma A, Agarwal V (2018). Rheumatology science and practice in India. Rheumatol Int.

[3] Malaviya AN, Shankar S, Arya V (2010). Indian Rheumatology Association consensus statement on the diagnosis and treatment of axial spondyloarthropathies. Indian J Rheumatol.

[4] Zahid-Al-Quadir A, Zaman MM, Ahmed S (2020). Prevalence of musculoskeletal conditions and related disabilities in Bangladeshi adults: a cross-sectional national survey. BMC Rheumatol.

[5] Rudwaleit MV, Van Der Heijde D, Landewé R (2019). Correction: the development of assessment of SpondyloArthritis International Society classification criteria for axial spondyloarthritis (part ii): validation and final selection. Ann Rheum Dis.

[6] Yi E, Ahuja A, Rajput T, George AT, Park Y (2020). Clinical, economic, and humanistic burden associated with delayed diagnosis of axial spondyloarthritis: a systematic review. Rheumatol Ther.

[7] Chorus AM, Boonen A, Miedema HS, van der Linden S (2002). Employment perspectives of patients with ankylosing spondylitis. Ann Rheum Dis.

[8] Baraliakos X, Berenbaum F, Favalli EG, Olivieri I, Ostendorf B, Poddubnyy D, DE Vlam K (2018). Challenges and advances in targeting remission in axial spondyloarthritis. J Rheumatol.

[9] Wang R, Ward MM (2018). Epidemiology of axial spondyloarthritis: an update. Curr Opin Rheumatol.

[10] (2025). Highlights of prescribing information | XELJANZ | Drugs@FDA: FDA-Approved Drugs. Highlights of prescribing information: XELJANZ®(tofacitinib)(package insert) [Internet.

[11] Maeshima K, Yamaoka K, Kubo S (2012). The JAK inhibitor tofacitinib regulates synovitis through inhibition of interferon-γ and interleukin-17 production by human CD4+ T cells. Arthritis Rheum.

[12] Burmester GR, Blanco R, Charles-Schoeman C (2013). Tofacitinib (CP-690,550 in combination with methotrexate in patients with active rheumatoid arthritis with an inadequate response to tumour necrosis factor inhibitors: a randomised phase 3 trial. Lancet.

[13] Kucharz EJ, Stajszczyk M, Kotulska-Kucharz A (2018). Tofacitinib in the treatment of patients with rheumatoid arthritis: position statement of experts of the Polish Society for Rheumatology. Reumatologia.

[14] Gladman D, Rigby W, Azevedo VF (2017). Tofacitinib for psoriatic arthritis in patients with an inadequate response to TNF inhibitors. N Engl J Med.

[15] Sandborn WJ, Su C, Sands BE (2017). Tofacitinib as induction and maintenance therapy for ulcerative colitis. N Engl J Med.

[16] Ruperto N, Brunner HI, Synoverska O (2021). Tofacitinib in juvenile idiopathic arthritis: a double-blind, placebo-controlled, withdrawal phase 3 randomised trial. Lancet.

[17] Deodhar A, Sliwinska-Stanczyk P, Xu H (2021). Tofacitinib for the treatment of ankylosing spondylitis: a phase III, randomised, double-blind, placebo-controlled study. Ann Rheum Dis.

[18] Gladman DD, Nash P ( 2024 ). Efficacy and safety of tofacitinib in an open-label, long-term extension study in patients with psoriatic arthritis who received adalimumab or tofacitinib in a Phase 3 randomized controlled study: a post hoc analysis. Arthritis Res Ther.

[19] Yesmin S, Abdal SJ (2018). Inflammatory back pain and associated disease conditions among patients with chronic low back pain in Bangladesh. Int J Rheum Dis.

[20] Abdal SJ, Yesmin S, Shazzad MN (2021). Development of a Bangla version of the Bath Ankylosing Spondylitis Disease Activity Index (BASDAI) and the Bath Ankylosing Spondylitis Functional Index (BASFI). Int J Rheum Dis.

[21] Islam N, Baron Basak T, OudeVoshaar MA, Ferdous N, Rasker JJ, Atiqul Haq S (2013). Cross-cultural adaptation and validation of a Bengali Health Assessment Questionnaire for use in rheumatoid arthritis patients. Int J Rheum Dis.

[22] Feroz AH, Islam MN, ten Klooster PM, Hasan M, Rasker JJ, Haq SA (2012). The Bengali Short Form-36 was acceptable, reliable, and valid in patients with rheumatoid arthritis. J Clin Epidemiol.

[23] Van der Heijde D (2018). Efficacy and safety of filgotinib, a selective Janus kinase 1 inhibitor, in patients with active ankylosing spondylitis (TORTUGA): results from a randomised, placebo-controlled, phase 2 trial. Lancet.

[24] Deodhar A, Van den Bosch F, Poddubnyy D (2022). Upadacitinib for the treatment of active non-radiographic axial spondyloarthritis (SELECT-AXIS 2): a randomised, double-blind, placebo-controlled, phase 3 trial. Lancet.

[25] Pope J, Sawant R, Tundia N (2020). Comparative efficacy of JAK inhibitors for moderate-to-severe rheumatoid arthritis: a network meta-analysis. Adv Ther.

[26] Lair-Mehiri L, Stefanescu C, Vaysse T (2020). Real-world evidence of tofacitinib effectiveness and safety in patients with refractory ulcerative colitis. Dig Liver Dis.

[27] Deodhar A, Akar S, Curtis JR (2024). Integrated safety analysis of tofacitinib from Phase 2 and 3 trials of patients with ankylosing spondylitis. Adv Rheumatol.

[28] Westhovens R (2019). Clinical efficacy of new JAK inhibitors under development. Just more of the same?. Rheumatology (Oxford).

[29] Navarro-Compán V, Deodhar A, Bahiri R, Bushmakin AG, Cappelleri JC, Rammaoui J (2024). Time to improvement of pain, morning stiffness, fatigue, and disease activity in patients with ankylosing spondylitis treated with tofacitinib: a post hoc analysis. Arthritis Res Ther.

[30] Ali N, Samadder M, Kathak RR, Islam F (2023). Prevalence and factors associated with dyslipidemia in Bangladeshi adults. PLoS One.

